# Curcumin–Coumarin Hybrid Analogues as Multitarget Agents in Neurodegenerative Disorders

**DOI:** 10.3390/molecules26154550

**Published:** 2021-07-28

**Authors:** Elías Quezada, Fernanda Rodríguez-Enríquez, Reyes Laguna, Elena Cutrín, Francisco Otero, Eugenio Uriarte, Dolores Viña

**Affiliations:** 1 Department of Organic Chemistry, Faculty of Pharmacy, Universidade de Santiago de Compostela, 15782 Santiago de Compostela, Spain; elias.quezada@usc.es (E.Q.); eugenio.uriarte@usc.es (E.U.); 2 Center for Research in Molecular Medicine and Chronic Disease (CIMUS), Department of Pharmacology, Pharmacy and Pharmaceutical Technology, Universidade de Santiago de Compostela, 15782 Santiago de Compostela, Spain; nana.enriquez@gmail.com (F.R.-E.); mdelosreyes.laguna@usc.es (R.L.); 3 Department of Pharmacy and Pharmaceutical Technology, Faculty of Pharmacy, Universidade de Santiago de Compostela, 15782 Santiago de Compostela, Spain; elena.cutrin@rai.usc.es (E.C.); francisco.otero@usc.es (F.O.); 4 Instituto de Ciencias Químicas Aplicadas, Universidad Autónoma de Chile, Santiago 7500912, Chile

**Keywords:** curcumin, curcumin–coumarin hybrids, neuroprotection, monoamine oxidase inhibition, cholinesterase inhibition, scavenging activity

## Abstract

Neurodegenerative diseases have a complex nature which highlights the need for multitarget ligands to address the complementary pathways involved in these diseases. Over the last decade, many innovative curcumin-based compounds have been designed and synthesized, searching for new derivatives having anti-amyloidogenic, inhibitory of tau formation, as well as anti-neuroinflammation, antioxidative, and AChE inhibitory activities. Regarding our experience studying 3-substituted coumarins with interesting properties for neurodegenerative diseases, our aim was to synthesize a new series of curcumin–coumarin hybrid analogues and evaluate their activity. Most of the 3-(7-phenyl-3,5-dioxohepta-1,6-dien-1-yl)coumarin derivatives **11**–**18** resulted in moderated inhibitors of hMAO isoforms and AChE and BuChE activity. Some of them are also capable of scavenger the free radical DPPH. Furthermore, compounds **14** and **16** showed neuroprotective activity against H_2_O_2_ in SH-SY5Y cell line. Nanoparticles formulation of these derivatives improved this property increasing the neuroprotective activity to the nanomolar range. Results suggest that by modulating the substitution pattern on both coumarin moiety and phenyl ring, ChE and MAO-targeted derivatives or derivatives with activity in cell-based phenotypic assays can be obtained.

## 1. Introduction

In neurodegenerative diseases, a loss of nerve cells is observed in the brain and spinal cord, leading to sensory dysfunction (dementia) or loss of function (ataxia). Mitochondrial dysfunction, oxidative stress, protein misfolding, neuroinflammation, and finally apoptosis have been recognized by different studies as pathological causes of neurodegenerative diseases such as Parkinson’s disease (PD), Alzheimer’s disease (AD), sclerosis multiple (MS), and amyotrophic lateral sclerosis (ALS). Currently, commercially available and approved drugs for these disorders only temporarily relieve symptoms but do not significantly alter disease progression. The development of new treatment strategies remains in the preclinical and clinical stages. Due to the complex nature of neurodegenerative diseases, it seems necessary to design multitarget ligands to address the complementary pathways involved in these diseases [1,2].

Curcumin is a dietary polyphenol presented in the curry spice turmeric. Numerous studies describe its therapeutic potential for neurodegenerative diseases, including AD and PD, due to its powerful antioxidant, anti-protein aggregation, and anti-inflammatory properties [3,4]. However, curcumin exhibits instability, poor bioavailability, and low cellular uptake, which limits the interest of its use in these disorders [5]. To address this problem, new nanoformulations such as liposomes, solid-lipid nanoparticles, micelles, polymer nanoparticles, and polymer conjugates have been developed [6,7]. With the same objective and also to improve its activity, in recent years, many compounds derived from curcumin have been designed and synthesized. Some of them have shown anti-amyloidogenic activity, inhibitory of the formation of tau, as well as anti-neuroinflammatory, antioxidant, and inhibitory of acetylcholinesterase (AChE) [8,9].

Coumarins are natural or synthetic compounds with diverse biological activities. Many synthetic coumarin derivatives have been designed to obtain new drugs with potential activity in neurodegenerative diseases. Coumarin moiety has the potential to achieve monoamine oxidase (MAO) inhibitory activity (e.g., MAO-A and MAO-B inhibitors), AChE, β- and γ-secretase inhibition. Some of these compounds display potent antioxidant activity and, therefore, could protect cells from neurodegeneration [10]. In the last few years, our group has described different series of 3-substituted coumarins displaying these properties [11,12,13,14,15,16,17].

MAO inhibition by coumarins may also prevent oxidative stress, through inhibition of neurotransmitters degradation, leading a neuroprotective effect. It has been described that systemic injection of a MAO inhibitor decreases 6-hydroxydopamine-induced oxidative stress [18]. 

**Figure 1:**
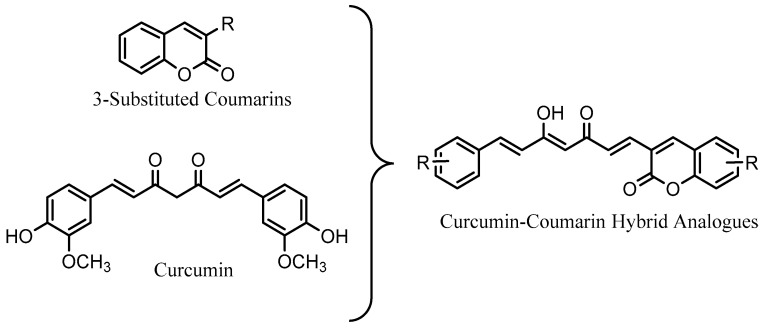
Overview of design of new curcumin–coumarin hybrid analogues.

Considering the above described and with the aim to improve the properties of curcumin and coumarin for the treatment of neurodegenerative diseases such as AD or PD, we have synthesized a series of curcumin–coumarin hybrid analogues (Figure 1) to study their activity as MAO and AChE inhibitors, free radical scavengers as well as their neuroprotective activity against hydrogen peroxide (H_2_O_2_). In addition, to facilitate their passage through cell membranes and therefore improve their neuroprotective activity, some of the derivatives have been formulated into nanoparticles.

## 2. Results

### 2.1. Synthesis of Coumarins ***5*** and ***9***

**Scheme 1:**
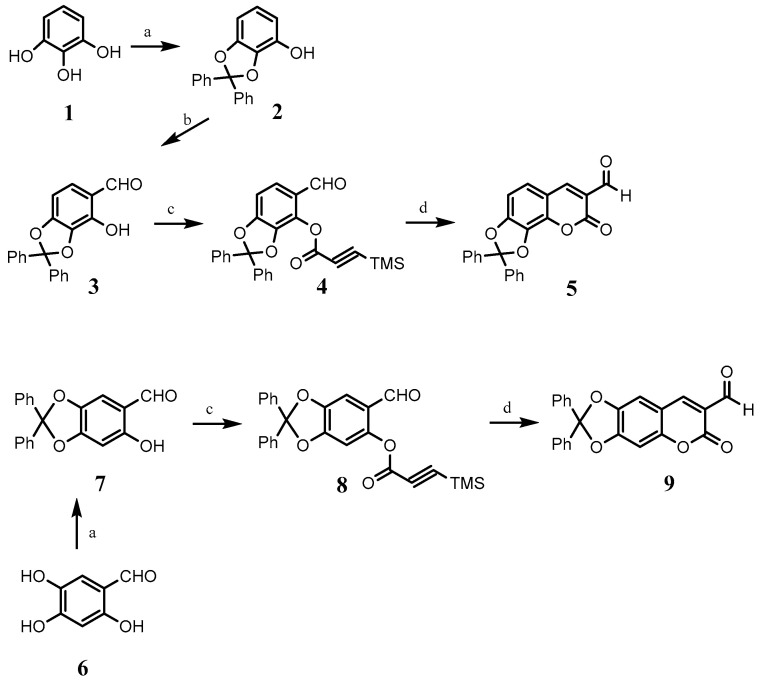
(a) dichlorodiphenylmethane, (Ph)_2_O, 180 °C, 30 min; (b) MgCl_2_, Et_3_N, (CH_2_O)_n_, THF reflux, 4 h; (c) (trimethylsilyl)propioloyl chloride, NaH, THF, reflux, 10 h; (d) DABCO, THF, reflux, 12 h.

For the synthesis of coumarin **5**, firstly, pyrogallol was treated with K_2_CO_3_ and dichlorodiphenylmethane in CH_3_CN, obtaining a protected catechol **2**. In a second step, the protected catechol was reacted with magnesium chloride, triethylamine, and *para*-formaldehyde to afford the protected *ortho*-hydroxybenzaldehyde **3**. Then, *ortho*-hydroxybenzaldehyde **3** was reacted with sodium hydride and (trimethylsilyl)propioloyl chloride to obtain the silylated ester **4**. Silylated ester **4** was reacted with 1,4-dizabicyclo[2.2.2]octane (DABCO) in THF under reflux, resulting in the desired coumarin **5** (Scheme 1).

Coumarin **9** was synthesized from 2,4,5-trihydroxybenzaldehyde (**6**) following a similar procedure to that described above (Scheme 1).

### 2.2. Synthesis of a Series of Curcumin–Coumarin Hybrid Analogues ***11***–***18***

**Scheme 2:**
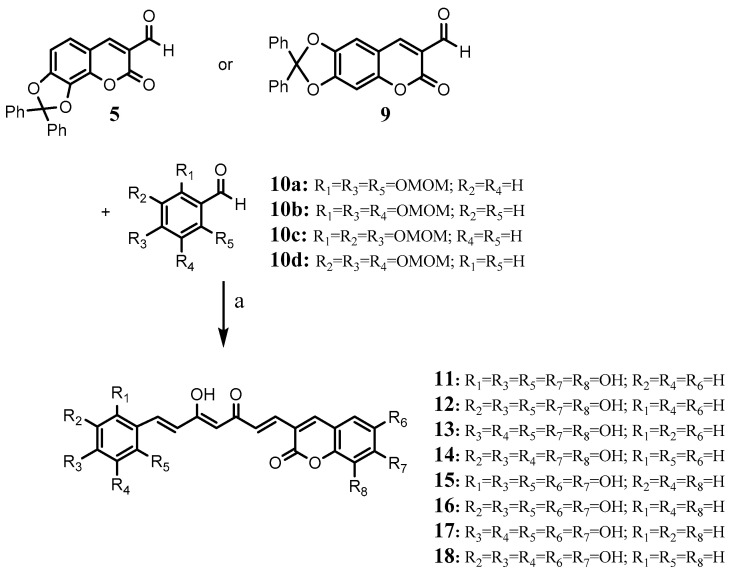
(a) i: 2,4-Pentanedione, B_2_O_3_, EtOAc, 40 °C, 2 h; (BuO)_3_B; nBuNH_2_, EtOAc, 25–40 °C 22 h; ii: HCl, 60 °C, 1 h; iii: H_2_, Pd/C, EtOH, rt, 48 h.

These compounds were obtained through direct coupling of an acethylacetone–B_2_O_3_ complex with the corresponding formylcoumarin previously obtained (**5** or **9**) and the adequate substituted benzaldehyde (**10a**–**d**) in the presence of tributyl borate and *n*-butylamine (Scheme 2). The deprotection of the phenol groups was carried out in two steps, firstly acidium medium was used to hydrolyze tris(methoxymethoxy) groups (OMOM) followed by hydrolysis of the diphenylbenzodioxole group to obtain compounds **11**–**18**. Compounds **11**–**18** were stored at −20 °C and in the dark.

### 2.3. Nanoparticles Formulations

Curcumin and curcumin–coumarin hybrid analogue loaded PLGA nanoparticles were prepared by an interfacial deposition method. All nanoparticles showed a narrow size distribution with mean diameters between 141–168 nm and PDI of 0.121–0.153, and a Zeta potential of −20 to −26 mV (dispersed in purified water). The encapsulation efficiency was similar for all the drugs assayed, obtaining percentages of encapsulation of 56, 53, and 55% for curcumin and curcumin–coumarin hybrid analogues **14** and **16,** respectively.

### 2.4. In Vitro Activity

#### 2.4.1. Cholinesterase Inhibition

As seen in Table 1, compounds **15** and **17** at 100 µM concentration inhibit the activity of both AChE and butyrylcholinesterase (BuChE) by approximately 50%. Therefore, their activity is lower than that presented by curcumin on AChE. Compound **12** resulted in the most selective derivative with activity only on BuChE.

#### 2.4.2. Monoamine Oxidase Inhibition

Most of the curcumin–coumarin hybrid analogues herein evaluated were not selective, inhibiting both MAO isoforms in a similar percentage (Table 1). The most potent inhibitor was compound **16** with IC_50_ (hMAO-B) = 26.18 ± 1.76 µM, which also exhibited greater selectivity over MAO-B. In any case, its inhibitory activity turned out to be lower than that shown by curcumin: IC_50_ (hMAO-A) = 10.18 ± 0.68 µM and IC_50_ (hMAO-B) = 1.78 ± 0.12 µM. For compounds **11**, **13**, **15**, **17**, their activity on the MAO isoforms could not be determined because they react with the Amplex Red reagent.

#### 2.4.3. Scavenging Activity

**Figure 2:**
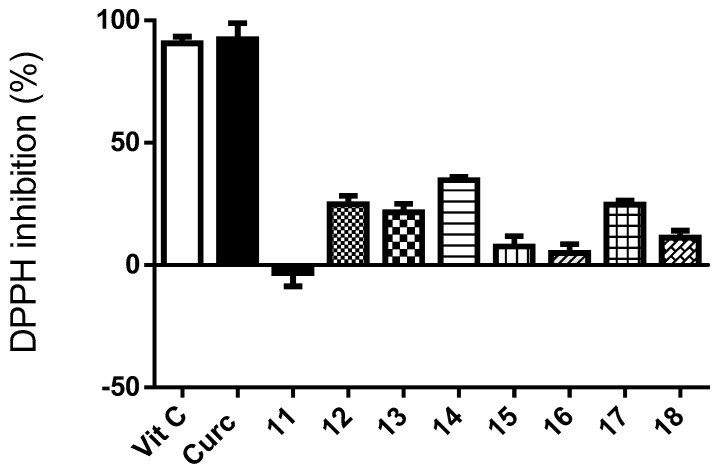
Percentage of neutralization of radical DPPH^.^ by curcumin, curcumin–coumarin hybrid analogues **11**–**18** (100 µM) and vitamin C used as reference (100 µM). Each value is the mean ± s.e.m. of 3 experiments (*n* = 3).

As seen in Figure 2, most of the curcumin–coumarin hybrid analogues **11**–**18** showed moderate activity as free radical scavengers. All of them resulted less active than curcumine or vitamin C. In general, among the curcumin–coumarin hybrid analogues, the compounds with the highest activity are those with three hydroxyl groups in contiguous positions of the phenyl substituent. Compounds **11**, **15,** and **16** that did not present this characteristic resulted in being the least active. 

#### 2.4.4. Neuroprotective Activity against H_2_O_2_

**Figure 3:**
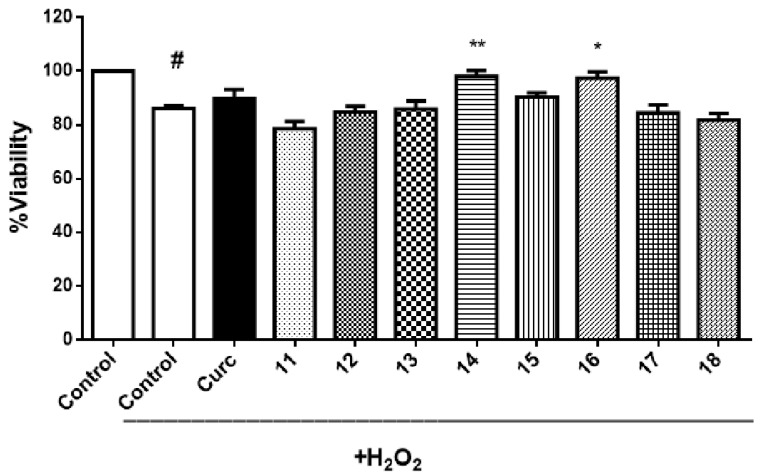
Neuroprotective effects of curcumin and curcumin–coumarin hybrid analogues **11**–**18** (10 µM) on SH-SY5Y cells. The results are expressed as % viability versus the control group (treated with DMSO 1%, or DMSO 1%, and H_2_O_2_ 100 µM). Each value is the mean ± s.e.m of at least five experiments. # *p* < 0.0001 versus the control group without H_2_O_2_ treatment. * *p* < 0.05, ** *p* < 0.005 versus DMSO + H_2_O_2_ treated group.

**Figure 4:**
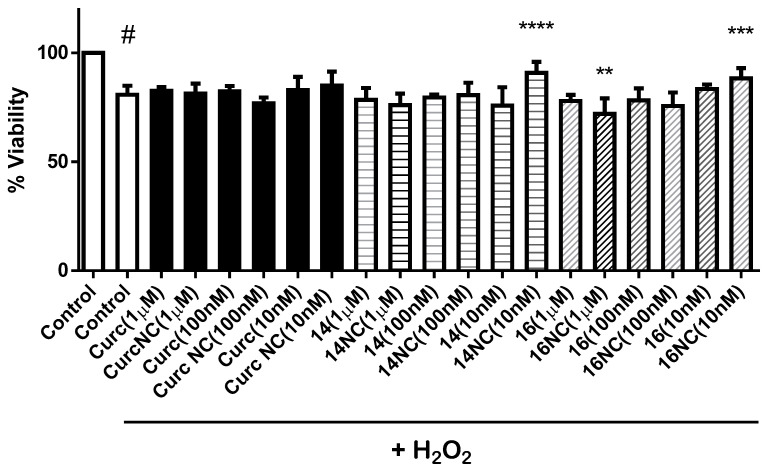
Neuroprotective effects on SH-SY5Y cells of different concentrations of curcumin and curcumin–coumarin hybrid analogues **14** and **16** and their nanoparticle formulations (NC). Each value is the mean ± s.e.m of at least 5 experiments. # *p* < 0.0001 versus the control group (without H_2_O_2_ treatment), ** *p* < 0.005, *** *p* < 0.001, **** *p* < 0.0001 versus cells treated with H_2_O_2_.

The neuroprotective activity of these curcumin–coumarin hybrid analogues **11**–**18** was evaluated in two different cell models, primary culture of rat motor cortex neurons and SH-SY5Y cell line. Neither curcumin nor any of the curcumin–coumarin hybrid analogues s **11**–**18** (10 µM) showed a protective effect against hydrogen peroxide (H_2_O_2_) in the primary culture of rat motor cortex (data not shown). However, **14** and **16** showed a significant increase of viability on the SH-SY5Y cell line treated with H_2_O_2_ (Figure 3). Because of this neuroprotective effect, compounds **14** and **16** were formulated in biodegradable nanoparticles, and their neuroprotective activity against H_2_O_2_ was also evaluated in the SH-SY5Y cell line. In this formulation, derivatives **14** and **16** at low concentration (10 nM) presented a statistically significant neuroprotective activity. As can be seen in Figure 4, the activity of compound **16** turned from a neurotoxic effect when the cultures were treated at 1 µM concentration to a neuroprotective effect at 10 nM concentration.

## 3. Discussion

Coumarins **5** and **9** were obtained by a similar route based on reactions described in the literature and both in good yield (68.3% and 81.3%, respectively). However, 3-(7-phenyl-3,5-dioxohepta-1,6-dien-1-yl)coumarin derivatives, namely as curcumin–coumarin hybrid analogues **11**–**18**, were obtained in low yields (5% approximately). This can be probably explained due to the low reactivity of the carbonyl of the formyl group at position 3 of coumarins. This fact was corroborated when obtaining in the same reaction the corresponding 1,7-biscoumarin-3,5-dioxohepta-1,6-dienyl derivatives in very low yield (data not shown). Furthermore, in the same reaction, we appreciated the formation of the corresponding 1,7-bisphenyl-3,5-dioxohepta-1,6-dienyl derivatives, previously described [19,20,21,22], and obtained in higher yields than the curcumin–coumarin hybrid analogues **11**–**18**. Despite the poor yield for curcumin–coumarin hybrid **11**–**18**, they could be easily detected and isolated because of their red color.

Regarding curcumin, the curcumin–coumarin hybrid analogues **11**–**18** conserve two aromatic systems in their structure, replacing a phenyl ring with a coumarin and maintain the length and flexibility of the central link region. These characteristics have been identified as a key for the derivatives to maintain the interest of curcumin in neurodegenerative diseases [23]. Additionally, different substitution partners on both aromatic systems, coumarin moiety, and phenyl ring have been studied.

MAO plays an important role in the homeostasis of neurotransmitters in the brain. MAO-B inhibitors are being used in combination with l-dopa to manage PD. However, the beneficial effects of MAO-B inhibitors in PD are not only associated with maintaining dopamine levels but also with their neuroprotective properties [24]. The occurrence of activated MAO-B in the brains of patients with AD has also been evidenced. Furthermore, MAO-A has a different appearance in different parts of the brains of patients with AD. MAO-A is increased in the hypothalamus and frontal pole, revealing that activated MAO-A in neurons is involved in the pathology of this disease as a predisposing factor. In addition, increased MAO-A activity appears more significant in the glia of patients with AD [25]. The above described demonstrates the interest in MAO inhibitors for the treatment of these diseases. Compounds **12**, **14**, **16,** and **18** showed moderate inhibitory activity on both MAO isoforms (Table 1). Hydroxyl substituents at positions 6 and 7 of the coumarin nucleus (compounds **16** and **18**) afforded more potent derivatives on the MAO-B isoform than substitution at positions 7 and 8 (compounds **12** and **14**). However, the position of the hydroxyl groups on the phenyl ring does not appear to significatively modify the activity of these compounds on MAO-B. The opposite behavior is observed in the activity of MAO-A. Compounds **12** and **16** resulted in the most potent derivatives, both bearing hydroxyl groups at positions 4, 6, and 7 of the phenyl ring. Only compound **16** exhibited moderate MAO-B selectivity [selectivity index (SI) = IC_50_ hMAO-A/IC_50_ hMAO-B; SI = 3.82].

Acetylcholine levels are regulated mainly by AChE but also by BuChE. Role of BuChE is less important than AChE in healthy brains. However, the AChE activity remains unchanged or even decreases in AD, while BuChE progressively increases, suggesting that inhibition of both enzymes may be considered a valid approach for AD therapy, increasing levels of AChE [26]. Curcumin–coumarin hybrid analogues **13**, **15,** and **17** showed similar activity on both AChE and BuChE, while compound **12** resulted in selectively inhibiting BuChE activity (Table 1). 

Among the studied curcumin–coumarin hybrid analogues, only compound **12** showed potential to inhibit both degradation of acetylcholine (via BuChE inhibition) and monoamines (via non-selective MAO inhibition) (Table 1).

Curcumin can protect neurons against inflammation, oxidative stress, apoptosis, or mitochondrial dysfunction [27,28]. It has been described that curcumin concentrations up to 20 µM increase viability in different cell models treated with H_2_O_2_ [29]. However, the effect of curcumin on SH-SY5Y cells is both dose and time-dependent. Approximately 40 µM concentration and 24 h exposure are the critical parameters at which the cell viability significantly decreases [30]. Other authors describe even lower concentrations (10 µM) to decreases SH-SY5Y proliferation and 20 µM to cause apoptosis [31]. Considering the controversies found in the literature, we studied the neuroprotective effects of low concentrations (≤10 µM) of curcumin and curcumin–coumarin hybrid analogues on two different neuronal models, primary culture of rat motor cortex neurons and SH-SY5Y cell line. While neither of the curcumin–coumarin hybrid analogues **11**–**18** nor curcumin protected rat motor cortex neurons against H_2_O_2_ (data not shown), compounds **14** and **16** showed neuroprotective effects at 10 µM concentration on SH-SY5Y cells (Figure 3). Compound **14** also showed scavenger activity (Figure 2) which could justify, at least partially, its neuroprotective activity. However, compound **16** lacks this activity, but it is the most potent MAO-B inhibitor, indicating that different mechanisms may be implicated in this neuroprotective activity.

Based on the statistically significant increase in viability on SH-SY5Y cells treated with H_2_O_2_ produced by compounds **14** and **16** at 10 µM concentration, both compounds were formulated in biodegradable nanoparticles. This formulation improves the neuroprotective effect at 10 nM concentration on SH-SY5Y cells treated with H_2_O_2_ (Figure 4). Furthermore, compound **16** formulated in nanoparticles goes from having a neurotoxic effect at 1 µM concentration to a statistically significant neuroprotective effect at 10 nM concentration. To explain this neuroprotective effect, it is necessary to resort to the hormesis, which is shared by several phytochemical compounds, including curcumin [32]. Hormesis is defined as a stimulation of cellular protection at low doses while it is inhibited at high doses of the compound, resulting in an inverted J or U-shaped dose-response curve as it can be obtained for compounds **14** and **16**. However, at concentrations used in this work, neuroprotective activity was not found for coumarin.

## 4. Materials and Methods

### 4.1. Materials and Instrumentation

All reactions utilizing air- or moisture-sensitive reagents were carried out in flame-dried glassware under an argon atmosphere, unless otherwise stated. Hexane, CH_2_Cl_2_, THF, Et_2_O, Et_3_N, and *n*-BuNH_2_ were distilled prior to use according to the standard protocols. Other reagents were purchased and used as received without further purification unless otherwise stated. Reactions were magnetically stirred and monitored by thin-layer chromatography (TLC). Analytical TLC was performed on plates precoated with silica gel (Merck 60 F254, 0.25 mm). Compounds were visualized with UV light and/or by staining with ethanolic phosphomolybdic acid (PMA) followed by heating on a hot plate. Flash chromatography (FC) was performed with silica gel (35–60 mesh) under pressure. Melting points were determined in a Reichert Kofler thermopan or in capillary tubes in a Buchi 510 apparatus and are uncorrected. NMR spectra were recorded on Bruker AMX 250 (^1^H, 250 MHz; ^13^C, 62.9 MHz) spectrometer in CDCl_3_ or DMSO-*d*_6_ with TMS as the internal standard. Chemical shifts (*δ*) are given in ppm and coupling constants (*J*) in Hz. Multiplicity is indicated as follows: s, singlet; d, doublet; m, multiplet; bs, broad singlet. Elemental analyses were performed on Thermo-Finnigan Flash 1112 CHNS/O analyzer (Supplementary Materials).

### 4.2. Chemical Synthesis

#### 4.2.1. Synthesis of 2,2-Diphenylbenzo[1,3]dioxol-4-ol (**2**) 

Dichlorodiphenylmethane (9.65 mL, 50.31 mmol) was added to a stirred mixture of pyrogallol (1, 4.23 g, 33.54 mmol) in diphenyl ether (25 mL), and the reaction mixture was heated at 180 °C for 30 min. The mixture was cooled to room temperature, and petroleum ether (50 mL) was added to give a solid compound [33,34]. Then the solid was filtered and purified by column chromatography using CH_2_Cl_2_ to yield **2** as a white solid (9.65 g, 99.2%). m.p.: 165 °C. ^1^H-NMR (250 MHz, CDCl_3_, δ ppm): 7.54 (4H, m), 7.37 (6H, m), 6.71 (1H, t, *J* = 6.7 Hz), 6.53 (1H, d, *J* = 6.4 Hz), 6.46 (1H, d, *J* = 6.4 Hz), 4.98 (1H, br). ^13^C-NMR (62.9 MHz, CDCl_3_, δ ppm): 148.2, 139.9, 139.3, 133.8, 129.1 (2C), 128.2 (4C), 126.3 (4C), 122.1, 116.1, 110.8, 101.9. Anal. Calcd. (%) for [C_19_H_14_O_3_]: C, 78.61; H, 4.86; found (%): C, 78.58; H, 4.83.

#### 4.2.2. Synthesis of 4-Hydroxy-2,2-diphenylbenzo[1,3]dioxol-5-carbaldehyde (**3**) 

To a dry THF solution (300 mL) of the 2,2-diphenylbenzo[1,3]dioxol-4-ol (**2**) (6.2 g, 21.35 mmol), anhydrous magnesium chloride (4.065 g, 42.70 mmol), triethylamine (5.95 mL, 4.32 g, 42.70 mmol) and paraformaldehyde (1.923 g, 64.05 mmol) were added. The reaction mixture was heated to reflux under Ar atmosphere for 4 h, and monitored by TLC (hexane:ethyl acetate = 8:2). After complete consumption of the phenol, the reaction mixture was cooled and diluted with diethyl ether (100 mL). The organic layer was washed successively with HCl (1 M, 2 × 100 mL) and H_2_O (2 × 100 mL), and then dried (Na_2_SO_4_) [35]. The product was purified by column chromatography using hexane:ethyl acetate (98:2) to yield **3** as a white solid (5.6 g, 82.5%). m.p.: 159 °C. ^1^H-NMR (250 MHz, CDCl_3_, δ ppm): 11.07 (1H, bs), 9.68 (1H, s), 7.59 (4H, m), 7.38 (6H, m), 7.12 (1H, d, *J* = 8.2 Hz), 6.62 (1H, d, *J* = 8.2 Hz). ^13^C-NMR (62.9 MHz, CDCl_3_, δ ppm): 194.9, 154.2, 145.3, 139.1 (2C), 133.6, 130.2, 129.3 (2C), 128.2 (4C), 126.0 (4C), 119.2, 118.09, 101.9. Anal. Calcd. (%) for [C_20_H_14_O_4_]: C, 75.46; H, 4.43; found (%): C, 75.44; H, 4.40.

#### 4.2.3. Synthesis of 5-Formyl-2,2-diphenylbenzo[1,3]dioxol-4-yle (trimethylsilyl)propiolate (**4**)

Sodium hydride (0.942 g, 23.55 mmol, 60%, washed with hexane) was suspended in anhydrous THF (25 mL), then cooled to 0 °C. A solution of **3** (2.50 g, 7.85 mmol) in anhydrous THF (25 mL) was dropwise added and the suspension was stirring for 1 h. Then, trimethylsilylpropioloyl chloride (3.78 g, 23.55 mmol) [36] in THF (10 mL) was dropwise added. The mixture was refluxed for 10 h. The mixture was quenched with ice-cold water and extracted three times with EtOAc. The combined organic layers were washed with brine, dried over Na_2_SO_4_, and evaporated [37]. The residue was purified by column chromatography on SiO_2_ hexane:ethyl acetate (2:98) to obtain silylated ester **4** as a colorless syrup (2.8 g, 80.7%). ^1^H-NMR (250 MHz, CDCl_3_, δ ppm): 9.93 (1H, s), 7.53 (4H, m), 7.39 (7H, m), 6.87 (1H, d, *J* = 8.2 Hz), 0.27 (9H, s). ^13^C-NMR (62.9 MHz, CDCl3, δ ppm): 186.8, 153.6, 149.1, 145.6, 139.5, 138.4 (2C), 129.5 (2C), 128.2 (4C), 127.1, 126.2 (4C), 123.5, 120.3, 106.8, 98.1, 92.8, −1.1. Anal. Calcd. (%) for [C_26_H_22_O_5_Si]: C, 70.57; H, 5.01; found (%): C, 70.56; H, 5.00.

#### 4.2.4. Synthesis of 2′,2′-Diphenyl-1,3-dioxol[h]coumarin-3-carbaldehyde (**5**) 

A mixture of silylated ester **4** (2.8 g, 6.33 mmol), and DABCO (1.42 g, 12.66 mmol) in THF (150 mL) was refluxed under Ar atmosphere. After 12 h, the mixture was diluted with CH_2_Cl_2_, washed with HCl (10%) and brine, and dried over Na_2_SO_4_ [38]. The solvent was evaporated to leave a residue, which was purified by silica-gel chromatography (CH_2_Cl_2_) to afford the 3-formylcoumarin **5** as a yellow solid (1.6 g, 68.3%). m.p.: 181 °C. ^1^H-NMR (250 MHz, CDCl_3_, δ ppm): 10.18 (1H, s), 8.31 (1H, s), 7.59 (4H, m), 7.40 (6H, m), 7.23 (1H, d, *J* = 8.3 Hz), 6.94 (1H, d, *J* = 8.3 Hz). ^13^C-NMR (62.9 MHz, CDCl_3_, δ ppm): 187.4, 159.3, 153.7, 146.2, 139.0, 138.6 (2C), 133.5, 129.6 (2C), 128.4 (4C), 126.4, 126.0 (4C), 120.6, 118.5, 114.3, 107.0. Anal. Calcd. (%) for [C_23_H_14_O_5_]: C, 74.59; H, 3.81; found (%): C, 74.56; H, 3.80.

#### 4.2.5. Synthesis of 6-Hydroxy-2,2-diphenylbenzo[1,3]dioxol-5-carbaldehyde (**7**) 

Following the procedure previously described to obtain compound **2**, dichlorodiphenylmethane (7.4 mL, 38.92 mmol) was reacted with **6** (4.0 g, 25.95 mmol) in diphenyl ether (25 mL), to yield **7** as a white solid (8.0 g, 96.8% yield) [33,39]. m.p.: 128 °C. ^1^H-NMR (250 MHz, CDCl_3_, δ ppm): 11.79 (1H, br), 9.59 (1H, s), 7.58 (4H, m), 7.39 (6H, m), 6.90 (1H, s), 6.55 (1H, s). ^13^C-NMR (62.9 MHz, CDCl_3_, δ ppm): 193.6, 161.4 (2C), 154.6, 141.0, 139.1 (2C), 129.4 (2C), 128.3 (4C), 126.1 (2C), 126.0 (2C), 109.4, 98.4, 90.1. Anal. Calcd. (%) for [C_20_H_14_O_4_]: C, 75.46; H, 4.43; found (%): C, 75.42; H, 4.42.

#### 4.2.6. Synthesis of 6-Formyl-2,2-diphenylbenzo[1,3]dioxol-5-yle(trimethylsilyl)propiolate (**8**) 

Following the procedure previously described to obtain compound **4**, compound **7** (2.5 g, 7.85 mmol) was reacted with sodium hydride (0.942 g, 23.55 mmol, 60%, washed with hexane), and trimethylsilylpropioloyl chloride (3.78 g, 23.55 mmol) [36] to obtain **8** as a colorless syrup (2.5 g, 72.0%) [37]. ^1^H-NMR (250 MHz, CDCl_3_, δ ppm): 9.96 (1H, s), 7.50 (5H, m), 7.34 (6H, m), 6.91 (1H, s), 0.20 (9H, s). ^13^C-NMR (62.9 MHz, CDCl_3_, δ ppm): 187.0, 154.3, 151.0, 145.9, 140.9, 138.2 (2C), 129.2 (2C), 128.4 (4C), 126.9 (4C), 125.8, 122.5, 118.2, 107.1, 97.9, 85.8. Anal. Calcd. (%) for [C_26_H_22_O_5_Si]: C, 70.57; H, 5.01; found (%): C, 70.55; H, 4.99.

#### 4.2.7. Synthesis of 2′,2′-Diphenyl-1,3-dioxol[g]coumarin-3-carbaldehyde (**9**) 

Following the procedure previously described to obtain compound **5**, a mixture of the propionic ester **8** (2.5 g, 5.65 mmol), and DABCO (1.26 g, 11.30 mmol) afforded the 3-formylcoumarin **9** as a yellow solid (1.7 g, 81.3%) [38]. m.p.: 173 °C. ^1^H-NMR (250 MHz, CDCl_3_, δ ppm): 9.97 (1H, s), 8.12 (1H, s), 7.44 (4H, m), 7.38 (6H, m), 6.92 (1H, s), 6.69 (1H, s). ^13^C-NMR (62.9 MHz, CDCl_3_, δ ppm): 191.8, 160.6, 152.1, 151.2, 145.3, 143.5, 138.9 (2C), 129.5 (2C), 128.4 (4C), 126.0 (4C), 122.4, 119.2, 108.3, 103.8, 98.4. Anal. Calcd. (%) for [C_23_H_14_O_5_]: C, 74.59; H, 3.81; found (%): C, 74.55; H, 3.79.

#### 4.2.8. Synthesis of 7,8-Dihydroxy-3-(7-(2′,4′,6′-trihydroxyphenyl)-3,5-dioxohepta-1,6-dien-1-yl)coumarin (**11**) 

2,4-Pentanedione (0.25 g, 2.5 mmol) and boric anhydride (0.121 g, 1.75 mmol) were dissolved in EtOAc (5 mL) and stirred for 2 h at 40 °C. Coumarin **5** (0.925 g, 2.5 mmol), tris(methoxymethoxy)benzaldehyde **10a** [40,41] (0.715 g, 2.5 mmol) and tributyl borate (2.3 g, 10 mmol) were added and the reaction mixture was stirred for 0.5 h. Then a solution of *n*-butylamine (0.182 g, 2.5 mmol) in EtOAc (2.5 mL) was dropwise added over a period of 30 min, the mixture was stirred for a further 18 h at room temperature and 4 h at 40 °C. The mixture was hydrolyzed by the addition of 0.4 N HCl (10 mL) and heating to 60 °C for 1 h. The organic layer was separated, and the aqueous layer was extracted three times with EtOAc. The combined organic layers were washed with water and dried over Na_2_SO_4_. Evaporation of the solvent left a red-brown powder [42]. The solid was dissolved in 80 mL of ethanol and treated with 10% Pd/C (0.6 g, 33 wt. % of starting material) [43]. The system was purged several times with hydrogen and stirred under hydrogen for 48 h. The reaction mixture was then purged with Ar and filtered through Celite washing with CH_3_OH. The filtrate was evaporated and the dark red solid was purified by column chromatography using CH_2_Cl_2_:CH_3_OH (9:1 and 8:2) to give **11** (44 mg, 4.15%). m.p.: 180 °C (dec.). ^1^H-NMR (250 MHz, DMSO-*d*_6_, δ ppm): 10.10 (2H, bs), 9.70 (2H, bs), 9.51 (2H, bs), 7.72 (1H, s), 7.46 (1H, d, *J* = 15.5 Hz), 7.15 (1H, d, *J* = 8.8 Hz), 6.84 (1H, d, *J* = 15.4 Hz), 6.64 (3H, m), 5.96 (2H, s), 5.83 (1H, s). ^13^C-NMR (62.9 MHz, DMSO-*d*_6_, δ ppm): 182.4, 169.7, 160.5, 158.7 (2C), 158.0, 148.7, 141.6, 139.5, 137.6, 134.0, 130.5, 126.9, 125.3, 120.7, 120.6, 114.3, 114.2, 109.9, 103.1, 95.7 (2C). Anal. Calcd. (%) for [C_22_H_16_O_9_]: C, 62.27; H, 3.80; found (%): C, 62.24; H, 3.78.

#### 4.2.9. Synthesis of 7,8-Dihydroxy-3-(7-(2′,4′,5′-trihydroxyphenyl)-3,5-dioxohepta-1,6-dien-1-yl)coumarin (**12**)

Following the procedure described above to obtain compound **11**, reaction of coumarin 5 (0.925 g, 2.5 mmol) and tris(methoxymethoxy)benzaldehyde **10b** [44] (0.715 g, 2.5 mmol) yielded compound **12** as a red solid (40 mg, 3.77%). m.p.: 177 °C (dec.). ^1^H-NMR (250 MHz, DMSO-*d*_6_, δ ppm): 10.12 (2H, bs), 9.76 (2H, bs), 9.50 (2H, bs), 7.79 (1H, d, *J* = 16.1 Hz), 7.65 (1H, s), 7.07 (1H, d, *J* = 8.8 Hz), 6.78 (1H, d, *J* = 15.4 Hz), 6.65 (1H, d, *J* = 16.1 Hz), 6.59 (2H, m), 6.42 (1H, s), 6.19 (1H, s), 5.83 (1H, s). ^13^C-NMR (62.9 MHz, DMSO-*d*_6_, δ ppm): 187.4, 179.5, 162.9, 152.5, 148.7, 148.6, 141.6, 140.4, 139.5, 137.6, 134.0, 129.0, 126.9, 124.2, 120.7, 120.6, 115.6, 114.4, 114.3, 114.2, 103.5, 96.9. Anal. Calcd. (%) for [C_22_H_16_O_9_]: C, 62.27; H, 3.80; found (%): C, 62.22; H, 3.77.

#### 4.2.10. Synthesis of 7,8-Dihydroxy-3-(7-(2′,3′,4′-trihydroxyphenyl)-3,5-dioxohepta-1,6-dien-1-yl)coumarin (**13**)

Following the procedure described above to obtain compound **11**, reaction of coumarin **5** (0.925 g, 2.5 mmol) and tris(methoxytrimethoxy)benzaldehyde **10c** [45,46] (0.715 g, 2.5 mmol) yielded compound **13** as a red solid (46 mg, 4.34%). m.p.: 185 °C (dec.). ^1^H-NMR (250 MHz, DMSO-*d*_6_, δ ppm): 10.10 (2H, bs), 9.74 (2H, bs), 9.52 (2H, bs), 7.79 (1H, d, *J* = 16.0 Hz), 7.72 (1H, s), 7.08 (1H, d, *J* = 8.8 Hz), 6.86 (1H, d, *J* = 15.8 Hz), 6.78 (1H, d, *J* = 16.1 Hz), 6.58 (2H, m), 6.51 (1H, d, *J* = 8.4 Hz), 6.12 (1H, d, *J* = 8.4 Hz), 5.78 (1H, s). ^13^C-NMR (62.9 MHz, DMSO-*d*_6_, δ ppm): 184.6, 162.4, 158.9, 148.8, 148.7, 147.4, 141.6, 139.5, 137.6, 134.0, 133.8, 128.4, 126.9, 124.4, 120.8, 120.7, 120.6, 114.7, 114.3, 114.2, 107.8, 101.6. Anal. Calcd. (%) for [C_22_H_16_O_9_]: C, 62.27; H, 3.80; found (%): C, 62.26; H, 3.79.

#### 4.2.11. Synthesis of 7,8-Dihydroxy-3-(7-(3′,4′,5′-trihydroxyphenyl)-3,5-dioxohepta-1,6-dien-1-yl)coumarin (**14**)

Following the procedure described above to obtain compound **11**, reaction of coumarin **5** (0.925 g, 2.5 mmol) and tris(methoxymethoxy)benzaldehyde **10d** [47,48,49] (0.715 g, 2.5 mmol) yielded compound **14** as a red solid (48 mg, 4.53%). m.p.: 183 °C (dec.). ^1^H-NMR (250 MHz, DMSO-*d*_6_, δ ppm): 10.09 (2H, bs), 9.70 (2H, bs), 9.53 (2H, bs), 7.70 (2H, m), 7.08 (1H, d, *J* = 8.8 Hz), 6.91 (1H, d, *J* = 16.1 Hz), 6.83 (1H, d, *J* = 16.1 Hz), 6.74 (1H, d, *J* = 15.8 Hz), 6.58 (1H, d, *J* = 8.8 Hz), 6.25 (2H, s), 5.85 (1H, s). ^13^C-NMR (62.9 MHz, DMSO-*d*_6_, δ ppm): 185.8, 180.1, 159.6, 148.7, 146.7 (2C), 141.6, 139.5, 137.6, 135.3, 135.1, 134.0, 128.9, 126.9, 123.0, 120.7, 120.6, 114.3, 114.2, 108.0 (2C), 101.9. Anal. Calcd. (%) for [C_22_H_16_O_9_]: C, 62.27; H, 3.80; found (%): C, 62.21; H, 3.78.

#### 4.2.12. Synthesis of 6,7-Dihydroxy-3-(7-(2′,4′,6′-trihydroxyphenyl)-3,5-dioxohepta-1,6-dien-1-yl)coumarin (**15**)

Following the procedure described above to obtain compound **11**, reaction of coumarin **9** (0.925 g, 2.5 mmol) and tris(methoxymethoxy)benzaldehyde **10a** (0.715 g, 2.5 mmol) yielded compound **15** as a red solid (50 mg, 4.72%). m.p.: 179 °C (dec). ^1^H-NMR (250 MHz, DMSO-*d*_6_, δ ppm): 10.09 (2H, bs), 9.75 (2H, bs), 9.51 (2H, bs), 7.77 (1H, d, *J* = 15.3 Hz), 7.58 (1H, s), 6.87 (1H, s), 6.75 (1H, d, *J* = 15.3 Hz), 6.60 (2H, m), 6.45 (1H, s), 5.97 (2H, s), 5.84 (1H, s). ^13^C-NMR (62.9 MHz, DMSO-*d*_6_, δ ppm): 185.2, 180.1, 160.5, 159.4, 158.7 (2C), 148.7, 148.1, 146.3, 139.2, 137.6, 130.5, 126.9, 125.3, 118.8, 113.7, 113.0, 104.9, 103.5, 102.1, 95.7 (2C). Anal. Calcd. (%) for [C_22_H_16_O_9_]: C, 62.27; H, 3.80; found (%): C, 62.25; H, 3.79.

#### 4.2.13. Synthesis of 6,7-Dihydroxy-3-(7-(2′,4′,5′-trihydroxyphenyl)-3,5-dioxohepta-1,6-dien-1-yl)coumarin (**16**)

Following the procedure described above to obtain compound **11**, reaction of coumarin **9** (0.925 g, 2.5 mmol) and tris(methoxytrimethoxy)benzaldehyde **10b** (0.715 g, 2.5 mmol) yielded compound **16** as a red solid (53 mg, 5.00%). m.p.: 188 °C (dec.). ^1^H-NMR (250 MHz, DMSO-*d*_6_, δ ppm): 10.10 (2H, bs), 9.76 (2H, bs), 9.55 (2H, bs), 7.75 (1H, d, *J* = 16.1 Hz), 7.67 (1H, s), 6.95 (1H, d, *J* = 15.5 Hz), 6.86 (1H, s), 6.68 (1H, d, *J* = 16.1 Hz), 6.53 (1H, d, *J* = 15.5 Hz), 6.38 (1H, s), 6.30 (1H, s), 6.15 (1H, s), 5.84 (1H, s). ^13^C-NMR (62.9 MHz, DMSO-*d*_6_, δ ppm): 183.8, 181.7, 159.1, 152.5, 148.7, 148.6, 148.1, 146.3, 140.4, 139.7, 137.6, 129.0, 126.9, 124.2, 118.8, 115.6, 114.3, 113.7, 113.0, 103.7, 102.2, 95.4. Anal. Calcd. (%) for [C_22_H_16_O_9_]: C, 62.27; H, 3.80; found (%): C, 62.20; H, 3.77.

#### 4.2.14. Synthesis of 6,7-Dihydroxy-3-(7-(2′,3′,4′-trihydroxyphenyl)-3,5-dioxohepta-1,6-dien-1-yl)coumarin (**17**)

Following the procedure described above to obtain compound **11**, reaction of coumarin **9** (0.925 g, 2.5 mmol) and tris(methoxytrimethoxy)benzaldehyde **10c** (0.715 g, 2.5 mmol) yielded compound **17** as a red solid (45 mg, 4.24%). m.p.: 190 °C (dec.). ^1^H-NMR (250 MHz, DMSO-*d*_6_, δ ppm): 10.05 (2H, bs), 9.70 (2H, bs), 9.45 (2H, bs), 7.75 (2H, m), 7.04 (1H, s), 6.95 (1H, d, *J* = 15.8 Hz), 6.71 (1H, d, *J* = 15.8 Hz), 6.57 (2H, m), 6.43 (1H, d, *J* = 8.4 Hz), 6.14 (1H, d, *J* = 8.4 Hz), 5.85 (1H, s). ^13^C-NMR (62.9 MHz, DMSO-*d*_6_, δ ppm): 186.4, 179.3, 159.0, 148.8, 148.7, 148.1, 147.4, 146.3, 139.2, 137.6, 133.8, 128.4, 126.9, 124.4, 120.8, 118.8, 114.7, 113.7, 113.0, 107.8, 102.1, 96.5. Anal. Calcd. (%) for [C_22_H_16_O_9_]: C, 62.27; H, 3.80; found (%): C, 62.24; H, 3.73.

#### 4.2.15. Synthesis of 6,7-Dihydroxy-3-(7-(3′,4′,5′-trihydroxyphenyl)-3,5-dioxohepta-1,6-dien-1-yl)coumarin (**18**)

Following the procedure described above to obtain compound **11**, reaction of coumarin **9** (0.925 g, 2.5 mmol) and tris(methoxytrimethoxy)benzaldehyde **10d** (0.715 g, 2.5 mmol) yielded compound **18** as a red solid (55 mg, 5.19%). m.p.: 185 °C (dec.). ^1^H-NMR (250 MHz, DMSO-*d*_6_, δ ppm): 10.12 (2H, bs), 9.77 (2H, bs), 9.53 (2H, bs), 7.78 (1H, d, *J* = 15.7 Hz), 7.69 (1H, s), 7.15 (1H, s), 6.94 (1H, d, *J* = 16.0 Hz), 6.75 (1H, d, *J* = 16.0 Hz), 6.55 (1H, d, *J* = 15.7 Hz), 6.45 (1H, s), 6.25 (2H, s), 5.84 (1H, s). ^13^C-NMR (62.9 MHz, DMSO-*d*_6_, δ ppm): 183.5, 181.8, 158.6, 148.7, 148.1, 146.7 (2C), 146.3, 139.2, 137.6, 135.3, 135.1, 128.9, 126.9, 123.0, 118.8, 113.7, 113.0, 108.0 (2C), 102.0, 94.6. Anal. Calcd. (%) for [C_22_H_16_O_9_]: C, 62.27; H, 3.80; found (%): C, 62.22; H, 3.73.

### 4.3. Formulation of Biodegradable Nanoparticles

Resomer^®^ RG503H (Evonic) polymer, which is a 50:50 copolymer of polylactic and polyglycolic acid in its acid form following the nanoprecipitation technique, was used to make the biodegradable nanoparticles [50]. Briefly, a 1% aqueous solution of poloxamer 407 (Sigma Aldrich) was used as the aqueous phase. Acetone was used as a solvent for the polymer and the active principles, the concentration of Resomer^®^ and drug being 0.4% and 0.1% (p/v), respectively. The organic phase was added slowly at room temperature, using a syringe, to the aqueous solution, under magnetic stirring. Finally, the acetone was removed by evaporation at 50 °C in a rotary evaporator (Buchi) until a final volume of 25 mL was obtained. Filtration was performed through 0.22 µm diameter polyamide membrane filters to remove the non-incorporated drug.

To determine the content of the active principle and the encapsulation efficiency, an aliquot of the nanosuspensions was diluted in ethanol to dissolve the Resomer^®^ and release the drugs, determining its concentration by spectrophotometry.

Size distribution (mean diameter and polydispersity index) and zeta potential of nanoparticles were determined in purified water at 25 °C using a Malvern Zetasizer Nano ZS instrument (Malvern Instruments Ltd., Malvern, UK).

### 4.4. Determination of hMAO-A and hMAO-B In Vitro Activity

The in vitro activity of the synthesized curcumin–coumarin hybrid analogues **11**–**18** or curcumin on hMAO enzymatic activity was evaluated using an Amplex^®^ Red MAO assay kit and following a fluorimetric method previously described by us [11]. Briefly, 50 µL of sodium phosphate buffer (0.05 M, pH 7.4) containing the test molecules (new compounds or reference inhibitors) in different concentrations and adequate amounts of recombinant hMAO-A or hMAO-B (adjusted to obtain in our experimental conditions the same reaction velocity (hMAO-A: 1.1 μg protein; specific activity: 150 nmol of *para*-tyramine oxidized to *para*-hydroxyphenylacetaldehyde/min/mg protein; hMAO-B: 7.5 μg protein; specific activity: 22 nmol of *para*-tyramine transformed/min/mg protein)) were incubated for 10 min at 37 °C in a flat-black bottom 96-well microtest plate, placed in the dark fluorimeter chamber. After this incubation period, the reaction was started by adding 50 µL of the mixture containing (final concentrations) 200 μM of the Amplex^®^ Red reagent, 1 U/mL of horseradish peroxidase and 1 mM of *para*-tyramine. The production of H_2_O_2_ and, consequently, of resorufin, was quantified at 37 °C in a multidetection microplate fluorescence reader (Fluo-star OptimaTM, BMG LABTECH, Offenburg, Germany) based on the fluorescence generated (excitation, 545 nm, emission, 590 nm) over a 10 min period, in which the fluorescence increased linearly. Control experiments were carried out simultaneously by replacing the tested molecules with appropriate dilutions of the vehicles. In addition, the possible capacity of these molecules to modify the fluorescence generated in the reaction mixture due to non-enzymatic inhibition (i.e., for directly reacting with Amplex^®^ Red reagent) was determined by adding these molecules to solutions containing only the Amplex^®^ Red reagent in sodium phosphate buffer. The specific fluorescence emission (used to obtain the final results) was calculated after subtraction of the background activity, which was determined from wells containing all components except the hMAO isoforms, which were replaced by sodium phosphate buffer solution. The IC_50_ values for each compound were calculated by linear regression representing the logarithm of the concentration (M) of the studied compound (abscissa axis) against the percentage of inhibition of the control MAO activity (ordinate axis). This linear regression was performed with 4–6 concentrations of each evaluated compound capable of inhibiting the control enzymatic activity of the MAO isoenzymes between 20% and 80%.

### 4.5. Determination of AChE and BuChE In Vitro Activity

Ellman’s method [51] was used to determine in vitro ChE activity. 0.01 U/mL human recombinant AChE expressed in HEK 293 cells or 0.0005 U/mL BuChE isolated from human serum were added to a 50 mM phosphate buffer solution (pH 7.2) containing different concentrations of curcumin–coumarin hybrid analogues **11**–**18** or curcumin. The mixture was preincubated at 37 °C for 5 min followed by the addition of 5 mM acetylthiocholine or butyrylthiocholine and 0.25 mM 5,5′-dithio-bis(2-nitrobenzoic acid) (DNTB). The activity was measured by the absorbance increasing at λ 412 nm at 1 min intervals for 10 min at 37 °C (Fluo-Star OptimaTM, BMG LABTECH, Offenburg, Germany). Control experiments were performed simultaneously by replacing the test drugs with appropriate dilutions of the vehicles. The specific absorbance (used to obtain the final results) was calculated after subtraction of the background activity, which was determined in wells containing all components except the AChE or BuChE, which was replaced by a sodium phosphate buffer solution.

### 4.6. DPPH Radical Scavenging Assay

The DPPH was dissolved in methanol (50 µM), and 99 µL of the solution was transferred to each well of a 96-well microplate. Curcumin–coumarin hybrid analogues **11**–**18**, coumarin or reference drug (vitamin C) were added to each well at a final concentration of 100 µM. Solutions were incubated at room temperature for 30 min. Absorbance was determined at λ 517 nm using a microplate reader (Fluo-star OptimaTM, BMG LABTECH, Offenburg, Germany). DPPH radical solution in methanol was used as a control, whereas a mixture of methanol and sample served as blank. The scavenging activity percentage (AA%) was determined according to the equation described by Mensor et al. [52]: AA% = 100 − ((Abssample − Absblank) × 100/Abscontrol)

### 4.7. Cell Culture

#### 4.7.1. Primary Culture of Rat Motor Cortex Neurons

Embryos were extracted by cesarean section from 18 days pregnant Wistar Kyoto rats which were euthanized by CO_2_ inhalation. Brains were carefully dissected out, and after removing meninges, a portion of the motor cortex was isolated. Fragments obtained from several embryos were mechanically digested and cells were resuspended in Neurobasal medium. The Neurobasal medium was supplemented with 2% B-27 to obtain cortex neuronal cultures. Cells were seeded in 96-well plates at a density of 200,000 cells/mL. Cultures were grown for 7–8 days in an incubator (Form Direct Heat CO_2_, Thermo Electron Corporation, Madrid, Spain) under saturated humidity at a partial pressure of 5% CO_2_ in air at 37 °C until a dense neuronal network could be observed [53].

#### 4.7.2. Human Neuroblastoma SH-SY5Y Cell Culture and Maintaining

The SH-SY5Y cells grew in a culture medium containing Ham’s F12 and MEM (mixture 1:1) and supplemented with 15% FBS, 1% L-Glutamine, 1% non-essential amino acids and 1% of penicillin G/streptomycin sulfate (all of them from Sigma-Aldrich S.A.) [54]. The cells were grown in 75 cm^2^ flasks in an incubator, under conditions of saturated humidity with a partial pressure of 5% CO_2_ in the air, at 37 °C. Cell culture medium was replaced every 2 days, and, at 80–90% of confluence, the cells were sub-cultured. To carry out the viability assays, the cells were seeded in sterile 96-well plates, with a density of 2 × 10^5^ cells/mL and grown distributed in aliquots of 100 μL for 24 h under the conditions described above.

#### 4.7.3. Cell Viability

Cells grown in 96-well plates were treated with H_2_O_2_ (100 µM) and curcumin or test compounds **11**–**18** (10 µM). When cells were treated with curcumin or curcumin–coumarin hybrid analogues **14** and **16** formulated in nanoparticles, they were added in the 24 h prior to H_2_O_2_ treatment. Then, cultures were incubated for 24 h. After this time, cell viability was determined using MTT (5 mg/mL in Hank’s). 10 μL of MTT solution was added to each well containing 100 μL of culture medium and the cells were incubated for 2 h as described above. Then, the culture medium was removed, 100 μL DMSO/well was added to solve the formazan crystals formed by the viable cells and the absorbance (λ 540 nm) was quantified in a plate reader. The viability (percentage) was calculated as (Absorbance (treatment)/Absorbance (negative control))100% [55]. Statistical analysis was performed using one way ANOVA test followed by Dunnett’s multiple comparison test by using GraphPad software.

## 5. Conclusions

A new series of curcumin–coumarin hybrid analogues **11**–**18** were synthesized in low yield starting from 2′,2′-diphenyl-1,3-dioxol[h]coumarin-3-carbaldehyde (**5**), or 2′,2′-diphenyl-1,3-dioxol[g]coumarin-3-carbaldehyde (**9**), and the corresponding tris(methoxymethoxy)benzaldehyde **10a**–**d**. Synthesized derivatives did not reach a potential as a multitarget drug. In general, they were either better at inhibiting MAO isoforms or AChE and BuChE activity. Only compound **12** inhibited BuChE and MAO isoforms with similar potency. In addition, compounds **14** and **16** resulted in being neuroprotective against H_2_O_2_ in SH-SY5Y cells. The formulation of these compounds in nanoparticles improves their neuroprotective activity at low concentrations. Results suggest that by modulating the substitution pattern on both coumarin moiety and phenyl ring, ChEs and MAO-targeted derivatives or derivatives with activity in cell-based phenotypic assays can be obtained.

## Figures and Tables

**Table 1:** Percentage inhibition of human cholinesterases (hAChE and hBuChE) and human monoamine oxidases (hMAO-A and hMAO-B).

Compound	hAChE % Inh 100 µM	hBuChE % Inh 100 µM	hMAO-A % Inh 100 µM	hMAO-B % Inh 100 µM
**11**	2.96% ± 0.10%	2.90% ± 0.10%	nd	nd
**12**	5.70% ± 0.40%	43.26% ± 2.90%	60.91% ± 4.09%	45.76% ± 3.07%
**13**	38.47% ± 2.58%	36.08% ± 2.42%	nd	nd
**14**	5.62% ± 0.40%	8.69% ± 0.60%	54.33% ± 3.64%	45.81% ± 3.07%
**15**	47.69% ± 3.20%	50.94% ± 3.41%	nd	nd
**16**	17.33% ± 1.16%	21.37% ± 1.43%	58.18% ± 3.90%	78.93% ± 5.59%
**17**	42.88% ± 2.87%	46.63% ± 3.12%	nd	nd
**18**	5.14% ± 0.34%	10.38% ± 0.70%	51.50% ± 3.45%	55.72% ± 3.74%
**Curcumin**	64.83% ± 4.34%	35.46% ± 2.38%	84.20% ± 5.61%	92.60% ± 6.17%

Results are expressed as the mean ± e.e.m (*n* = 3). nd: not determined. At concentration > 100 µM, compounds precipitate.

## Data Availability

Not applicable.

## Supplementary Materials

The following are available online. 1H and 13C NMR of compounds **5**, **9**, **10a**–**d**, **11**–**18**.

## Sample Availability

Samples of the compounds **5**, **9**, **10a**–**d**, **11**–**18** are available from the authors.

## References

[1] Gabr M.T., Yahiaoui S. (2020). Multitarget therapeutics for neurodegenerative diseases. BioMed Res. Int..

[2] Ramsay R.R., Majekova M., Medina M., Valoti M. (2016). Key targets for multi-target ligands designed to combat neurodegeneration. Front. Neurosci..

[3] Eghbaliferiz S., Farhadi F., Barreto G.E., Majeed M., Sahebkar A. (2020). Effects of cucrcumin on neurological diseases: Focus on astrocytes. Pharmacol. Rep..

[4] Abass S., Latif M.S., Shafie N.S., Ghazali M.I., Kormin F. (2020). Neuroprotective expression of turmeric and curcumin. Food Res..

[5] Nelson K.M., Dahlin J.L., Bisson J., Graham J., Pauli G.F., Walters A. (2017). The essential medicinal chemistry of curcumin. J. Med. Chem..

[6] Gera M., Sharma N., Ghosh M., Luong H.D., Lee S.L., Min T., Kwon T., Jeong D.K. (2017). Nanoformulations of curcumin: An emerging paradigm for improved remedial application. Oncotarget.

[7] Salehi B., Calina D., Docea A.O., Koirala N., Aryal S., Lombardo D., Pasqua L., Taheri Y., Salgado Castillo C.M., Martorell M. (2020). Curcumin’s nanomedicine formulations for therapeutic application in neurological diseases. J. Clin. Med..

[8] Chainoglou E., Hadjipavlou-Litina D. (2020). Curcumin in health and diseases: Alzheimer’s disease and curcumin analogues, derivatives, and hybrids. Int. J. Mol. Sci..

[9] Lo Cascio F., Marzullo P., Kayed R., Palumbo Piccionello A. (2021). Curcumin as scaffold for drug discovery against neurodegenerative diseases. Biomedicines.

[10] Jameel E., Umar T., Kumar J., Hoda N. (2016). Coumarin: A privileged scaffold for the design and development of antineurodegenerative agents. Chem. Biol. Drug Des..

[11] Matos M.J., Terán C., Pérez-Castillo Y., Uriarte E., Santana L., Viña D. (2011). Synthesis and study of a series of 3-arylcoumarins as potent and selective monoamine oxidase B inhibitors. J. Med. Chem..

[12] Matos M.J., Vilar S., Gonzalez-Franco R.M., Uriarte E., Santana L., Friedman C., Tatonetti N.P., Viña D., Fontenla J.A. (2013). Novel (coumarin-3-yl)carbamates as selective MAO-B inhibitors: Synthesis, in vitro and in vivo assays, theoretical evaluation of ADME properties and docking study. Eur. J. Med. Chem..

[13] Matos M.J., Rodríguez-Enríquez F., Borges F., Santana L., Uriarte E., Estrada M., Rodríguez-Franco M.I., Laguna R., Viña D. (2015). 3-Amidocoumarins as potential multifunctional agents against neurodegenerative diseases. ChemMedChem.

[14] Delogu G., Picciau C., Ferino G., Quezada E., Podda G., Uriarte E., Viña D. (2011). Synthesis, human monoamine oxidase inhibitory activity and molecular docking studies of 3-heteroarylcoumarin derivatives. Eur. J. Med. Chem..

[15] Costas-Lago M.C., Besada P., Rodríguez-Enríquez F., Viña D., Vilar S., Uriarte E., Borges F., Terán C. (2017). Synthesis and structure-activity relationship study of novel 3-heteroarylcoumarins based on pyridazine scaffold as selective MAO-B inhibitors. Eur. J. Med. Chem..

[16] Matos M.J., Herrera Ibatá D.M., Uriarte E., Viña D. (2020). Coumarin-rasagiline hybrids as potent and selective hMAO-B inhibitors, antioxidants, and neuroprotective agents. ChemMedChem.

[17] Rodríguez-Enríquez F., Costas-Lago M.C., Besada P., Alonso-Pena M., Torres-Terán I., Viña D., Fontenla J.Á., Sturlese M., Moro S., Quezada E. (2020). Novel coumarin-pyridazine hybrids as selective MAO-B inhibitors for the Parkinson’s disease therapy. Bioorg. Chem..

[18] Finberg J.P.M., Rabey J.M. (2016). Inhibitors of MAO-A and MAO-B in psychiatry and neurology. Front Pharmacol..

[19] Zuo Y., Huang J., Zhou B., Wang S., Shao W., Zhu C., Lin L., Wen G., Wang H., Du J. (2012). Synthesis, cytotoxicity of new 4-arylidene curcumin analogues and their multi-functions in inhibition of both NF-κB and Akt signalling. Eur. J. Med. Chem..

[20] Lin L., Shi Q., Nyarko A.K., Bastow K.F., Wu C.C., Su C.Y., Shih C.C.Y., Lee K.H. (2006). Antitumor Agents. 250. Design and synthesis of new curcumin analogues as potential anti-prostate cancer agents. J. Med. Chem..

[21] Lee K.H., Lin L., Shih C.C.Y., Su C.Y., Ishida J., Ohtsu H., Wang H.-K., Itokawa H., Chang C. (2008). Curcumin Analogues and uses Thereof. U.S. Patent.

[22] Qiu X., Du Y., Lou B., Zuo Y., Shao W., Huo Y., Huang J., Yu Y., Zhou B., Du J. (2010). Synthesis and identification of new 4-arylidene curcumin analogues as potential anticancer agents targeting nuclear factor-κB signaling pathway. J. Med. Chem..

[23] Reinke A.A., Gestwicki J.E. (2007). Structure-activity relationships of amyloid beta-aggregation inhibitors based on curcumin: Influence of linker length and flexibility. Chem. Biol. Drug Des..

[24] Finberg J.P.M. (2014). Update on the pharmacology of selective inhibitors of MAO-A and MAO-B: Focus on modulation of CNS monoamine neurotransmitter release. Pharmacol. Therapeut..

[25] Cai Z. (2014). Monoamine oxidase inhibitors: Promising therapeutic agents for Alzheimer’s disease. Mol. Med. Report..

[26] Lane R.M., Potkin S.G., Enz A. (2006). Targeting acetylcholinesterase and butyrylcholinesterase in dementia. Int. J. Neuropsychopharmacol..

[27] Kandezi K., Mohammadi M., Ghaffari M., Gholami M., Motaghinejad M., Safari S. (2020). Novel insight to neuroprotective potential of curcumin: A mechanistic review of possible involvement of mitochondrial biogenesis and PI3/Aky/GSK3 or PI3/Akt/CREB/BDNF signaling pathways. Int. J. Mol. Cell. Med..

[28] Sandoval-Avila S., Diaz N.F., Gómez-Pinedo U., Canales-Aguirre A.A., Gutiérrez-Mercado Y.K., Padilla-Camberos E., Marquez-Aguirre A.L., Díaz-Martínez N.E. (2019). Neuroprotective effects of phytochemicals on dopaminergic neuron cultures. Neurología.

[29] Mufti S., Bautista A., Pino-Figueroa A. (2015). Evaluation of the neuroprotective effects of curcuminoids on B35 and SH-SY5Y neuroblastoma cells. Med. Aromat. Plants.

[30] Yu X., Chen L., Tang M., Yang Z., Fu A., Wang Z., Wang H. (2019). Revealing the effects of curcumin on SH-SY5Y neuronal cells: A combined study from cellular viability, morphology, and biomechanics. J. Agric. Food Chem..

[31] Zhai K., Brockmüller A., Kubatka P., Shakibaei M., Büsselberg D. (2020). Curcumin’s beneficial effects on neuroblastoma: Mechanisms, challenges, and potential solutions. Biomolecules.

[32] Moghaddam N.S.A., Oskouie M.N., Butler A.E., Petit P.X., Barreto G.E., Sahebkar A. (2019). Hormetic effects of curcumin: What is the evidence?. J. Cell Physiol..

[33] Kim M.K., Choo H., Chong Y. (2014). Water-soluble and cleavable quercetin–amino acid conjugates as safe modulators for P-glycoprotein-based multidrug resistance. J. Med. Chem..

[34] Casagrande C., Ferrini R., Miragoli G., Ferrari G. (1973). β-Adrenergic receptor inhibitors. IV. 1-(Hydroxy and dihydroxyphenoxy)-3-isopropylamine-2-propanols. Boll. Chim. Farm..

[35] Akselsen O.W., Skattebøl L., Hansen T.V. (2009). ortho-Formylation of oxygenated phenols. Tetrahedron Lett..

[36] Medvedeva A.S., Andreev M.V., Safronova L.P., Afonin A.V. (2005). Synthesis of trimethylsilylpropynoyl chloride. Russ. J. Org. Chem..

[37] Nakagawa-Goto K., Lee K. (2006). Anti-AIDS agents 68. The first total synthesis of a unique potent anti-HIV chalcone from genus Desmos. Tetrahedron Lett..

[38] Matsuya Y., Hayashi K., Nemoto H. (2005). A new protocol for the consecutive a- and b-activation of propiolates towards electrophiles, involving conjugate addition of tertiary amines and intramolecular silyl migration. Chem. Eur. J..

[39] Pereira A.R. (2007). Marine Natural Products: Synthesis and Isolation of Bioactive Analogues. Ph.D. Thesis.

[40] Chen D., Chen R., Wang R., Li J., Xie K., Bian C., Sun L., Zhang X., Liu J., Yang L. (2015). Probing the catalytic promiscuity of a regio- and stereospecific C-glycosyltransferase from Mangifera indica. Ang. Chem. Int. Ed..

[41] Chao S.W., Su M.Y., Chiou L.C., Chen L.C., Chang C.I., Huang W.J. (2015). Total synthesis of hispidulin and the structural basis for its inhibition of proto-oncogene kinase Pim-1. J. Nat. Prod..

[42] Mazumder A., Neamati N., Sunder S., Schulz J., Pertz H., Eich E., Pommier Y. (1997). Curcumin analogs with altered potencies against HIV-1 integrase as probes for biochemical mechanisms of drug action. J. Med. Chem..

[43] Feldman K.S., Lawlor M.D. (2000). Ellagitannin Chemistry. The first total synthesis of a dimeric ellagitannin, coriariin A. J. Am. Chem. Soc..

[44] Xu J., Wang H., Sim M.M. (2003). First synthesis of (±)-C-3-prenylated flavanones. Synth. Commun..

[45] Teng Y., Li X., Yang K., Li X., Zhang Z., Wang L., Deng Z., Song B., Yan Z., Zhang Y. (2017). Synthesis and antioxidant evaluation of desmethylxanthohumol analogs and their dimers. Eur. J. Med. Chem..

[46] Hofmann E., Webster J., Do T., Kline R., Snider L., Hauser Q., Higginbottom G., Campbell A., Ma L., Paula S. (2016). Hydroxylated chalcones with dual properties: Xanthine oxidase inhibitors and radical scavengers. Bioorg. Med. Chem..

[47] Hirotaka E., Naoko Y. (2019). Adhesive composition containing copolymer having gallol group-like side chain and method for producing the copolymer. Jpn. Kokai Tokkyo Koho.

[48] Zhan K., Sung K., Ejima H. (2017). Tunicate-inspired gallol polymers for underwater adhesive: A comparative study of catechol and gallol. Biomacromolecules.

[49] Roschek B., Fink R.C., McMichael M.D., Li D., Alberte R.S. (2009). Elderberry flavonoids bind to and prevent H1N1 infection in vitro. Phytochemistry.

[50] Lopalco A., Ali H., Denora N., Rytting E. (2015). Oxcarbazepine-loaded polymeric nanoparticles: Development and permeability studies across in vitro models of the blood-brain barrier and human placental trophoblast. Int. J. Nanomed..

[51] Ellman G.L., Counrtney K.D., Andres V., Featherstone R.M.A. (1961). A new and rapid colorimetric determination of acetylcholinesterase activity. Biochem. Pharmacol..

[52] Mensor L.L., Menezes F.S., Leitão G.G., Reis A.S., dos Santos T.C., Coube C.S., Leitão S.G. (2001). Screening of Brazilian plant extracts for antioxidant activity by the use of DPPH free radical method. Phytother. Res..

[53] Yáñez M., Matías-Guiu J., Arranz-Tagarro J.-A., Galán L., Viña D., Gómez-Pinedo U., Vela A., Guerrero A., Martínez-Vila E., García A.G. (2014). The neuroprotection exerted by memantine, minocycline and lithium, against neurotoxicity of CSF from patients with amyotrophic lateral sclerosis, is antagonized by riluzole. Neurodegen. Dis..

[54] Xicoy H., Wiering B., Martens G.J.M. (2017). SH-SY5Y cell line in Parkinson’s disease research: A systematic review. Mol. Neurodegener..

[55] Liu Y., Peterson D.A., Kimura H., Schubert D. (1997). Mechanism of cellular 3-(4,5-dimethylthiazol-2-yl)-2,5-diphenyltetrazolium bromide (MTT) reduction. J. Neurochem..

