# Supplementary material for: Curcumin–Coumarin Hybrid Analogues as Multitarget Agents in Neurodegenerative Disorders
Source: Molecules. 2021 Jul 28;26(15):4550. doi: 10.3390/molecules26154550 (PMC8348017; doi:10.3390/molecules26154550)

# Supporting information

## Curcumin-Coumarin Hybrid Analogues as Multitarget Agents in Neurodegenerative Disorders

**Elías Quezada[1], Fernanda Rodríguez-Enríquez[2], Reyes Laguna[2], Elena Cutrín[3], Francisco Otero[3], Eugenio Uriarte[1,4] and Dolores Viña[2,*]**

[1] Department of Organic Chemistry, Faculty of Pharmacy, Universidade de Santiago de Compostela, 15782 Santiago de Compostela, Spain; elias.quezada@usc.es; eugenio.uriarte@usc.es

[2] Department of Pharmacology, Pharmacy and Pharmaceutical Technology and Center for Research in Molecular Medicine and Chronic Disease (CIMUS), Universidade de Santiago de Compostela, 15782 Santiago de Compostela, Spain; nana.enriquez@gmail.com; mdelosreyes.laguna@usc.es

[3] Department of Pharmacy and Pharmaceutical Technology, Faculty of Pharmacy, Universidade de Santiago de Compostela, 15782 Santiago de Compostela, Spain; elena.cutrin@rai.usc.es; francisco.otero@usc.es

[4] Instituto de Ciencias Químicas Aplicadas, Universidad Autónoma de Chile, 7500912 Santiago, Chile

*Correspondence: mdolores.vina@usc.es; Tel.: +34 881 815 424

**Compound 5**: $^{1}$H and $^{13}$C NMR

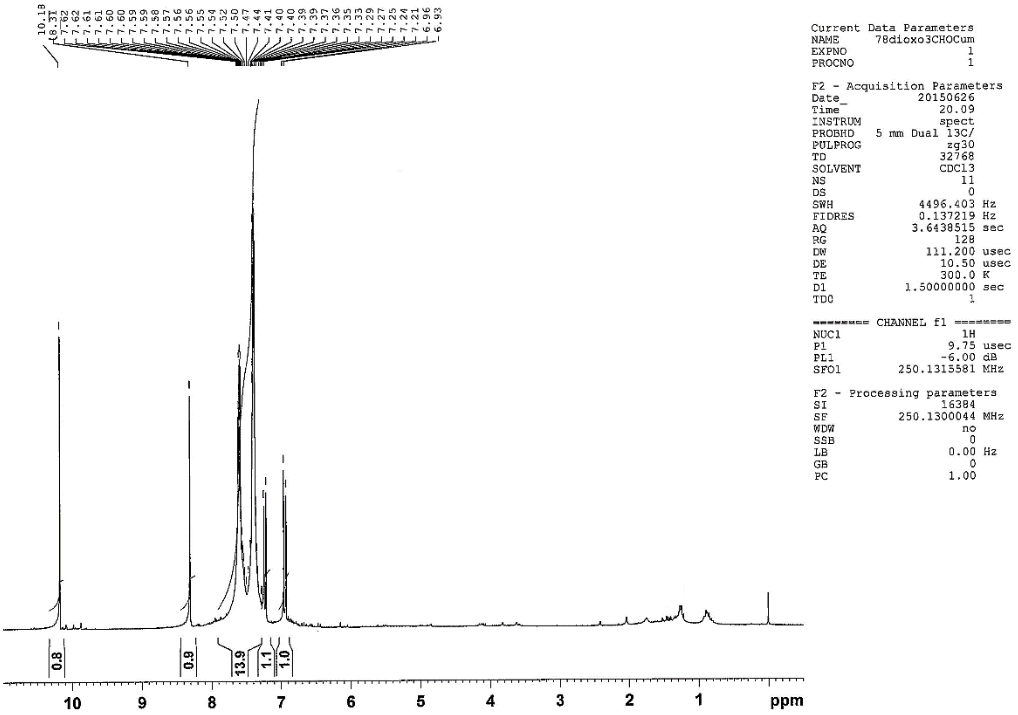


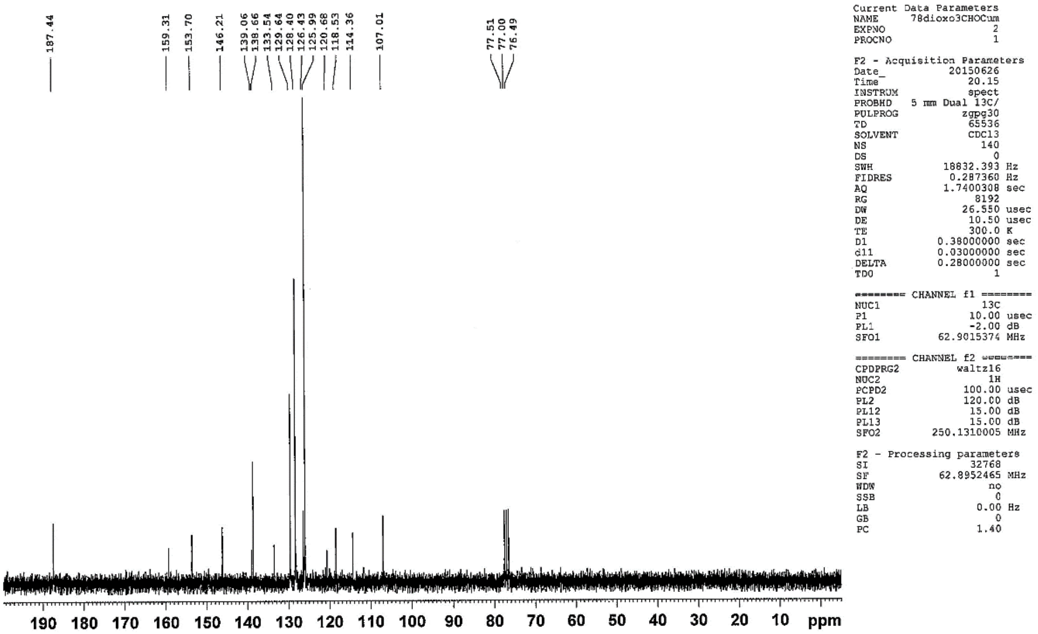

**Compound 9**: $^{1}$H and $^{13}$C NMR

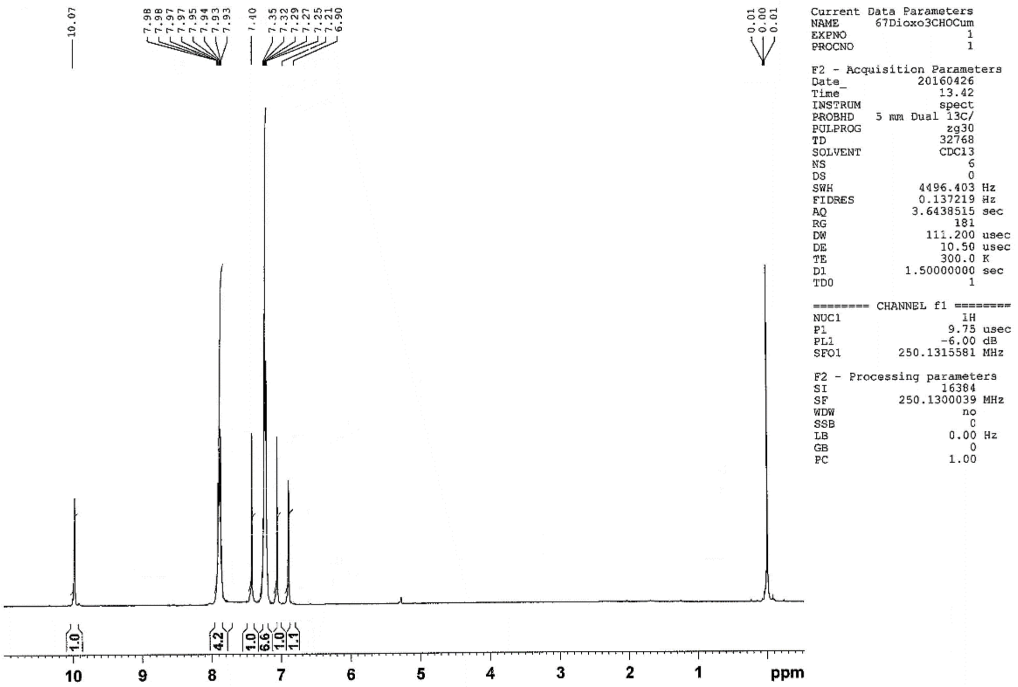


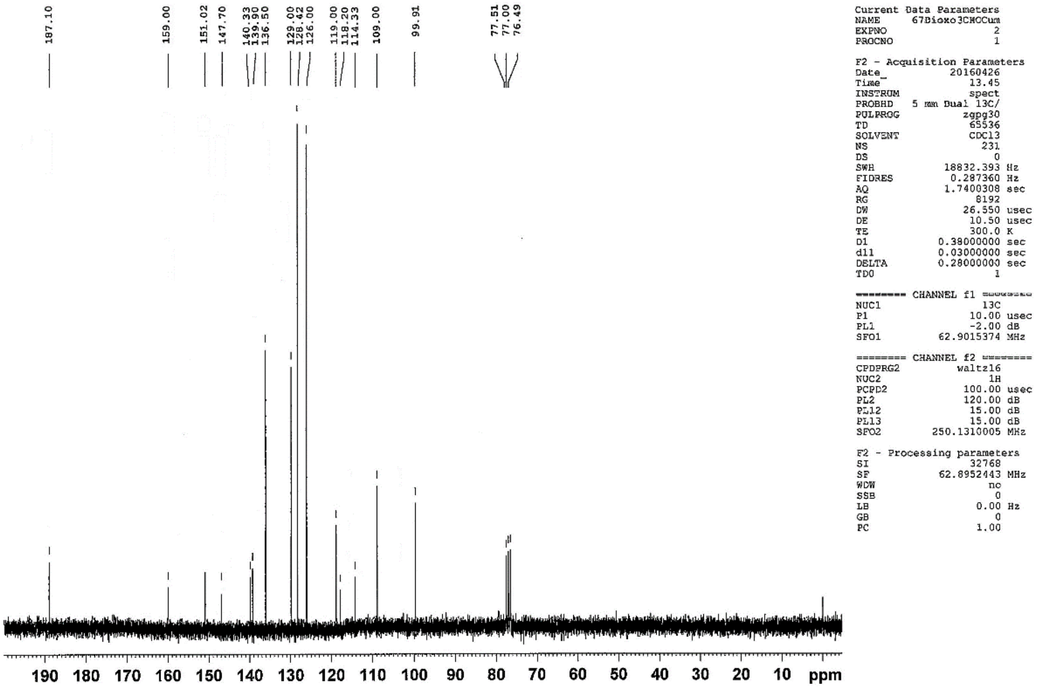

**Compound 11**: $^{1}$H and $^{13}$C NMR

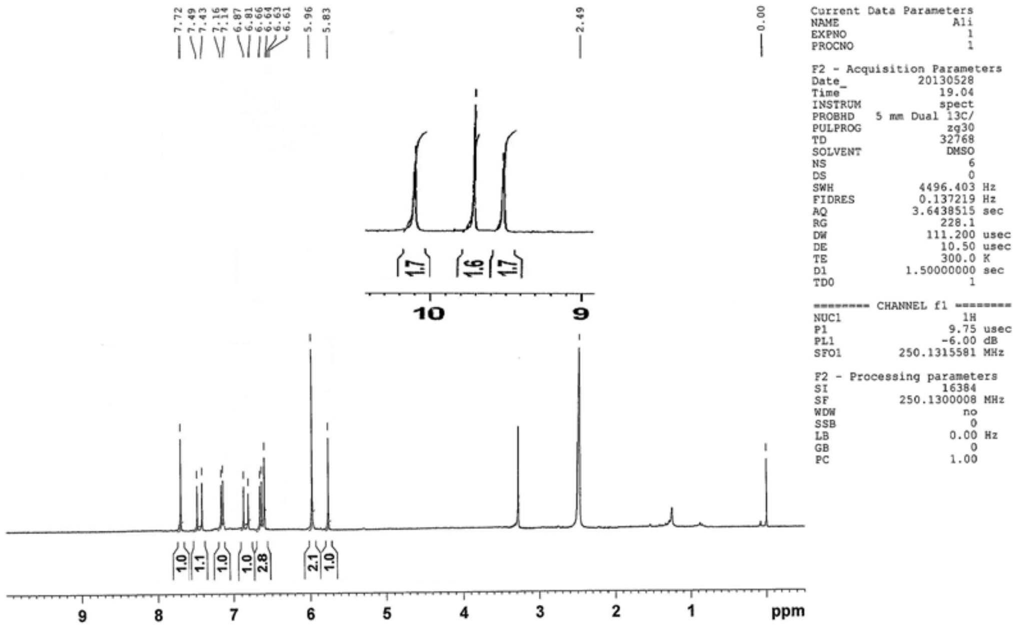


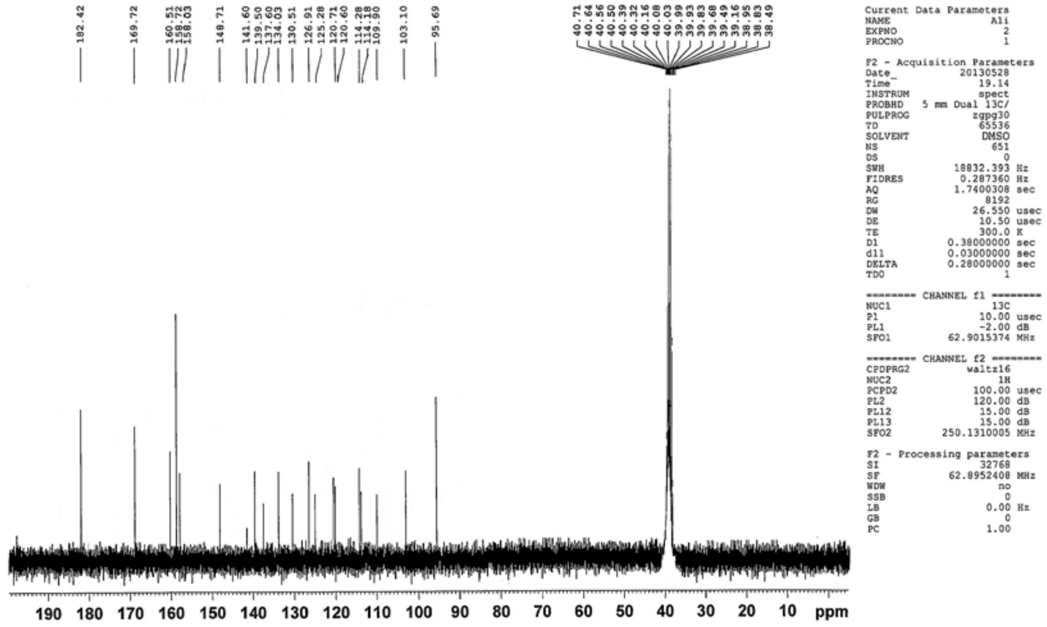

**Compound 12**: $^{1}$H and $^{13}$C NMR

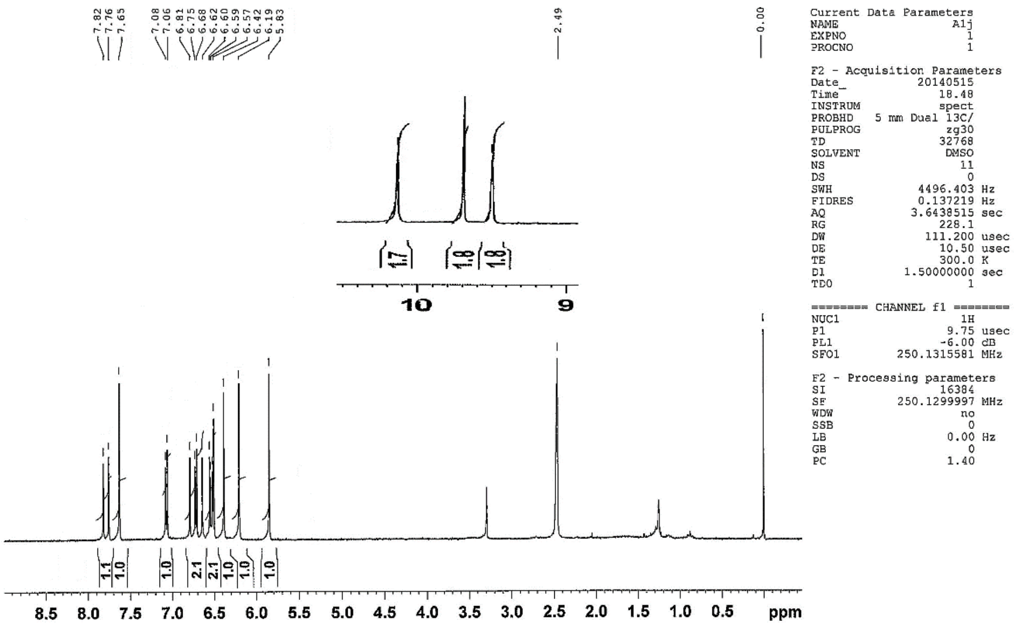

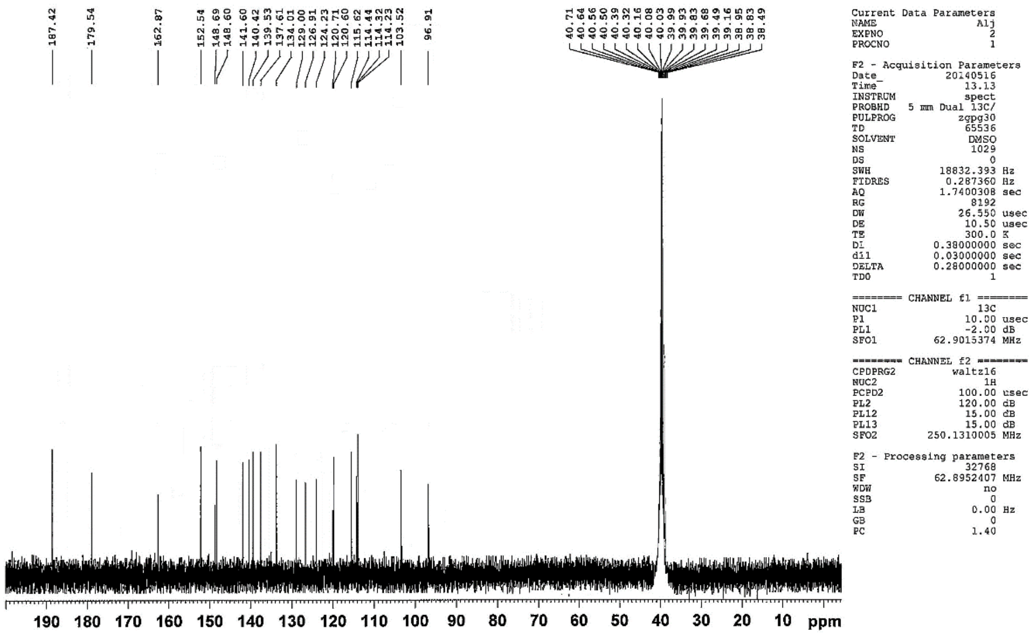

**Compound 13**: $^{1}$H and $^{13}$C NMR

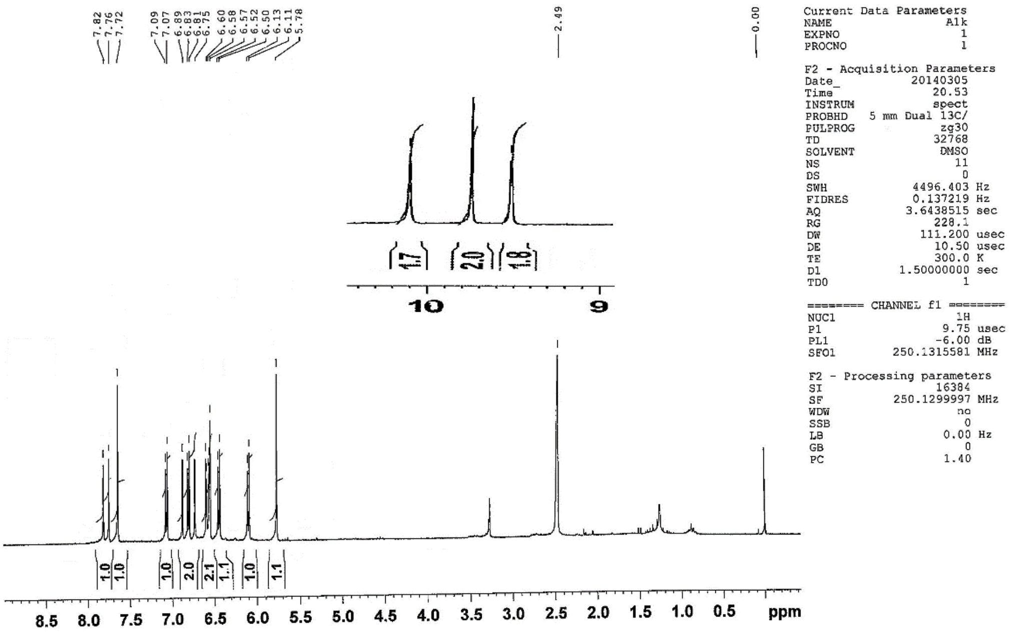


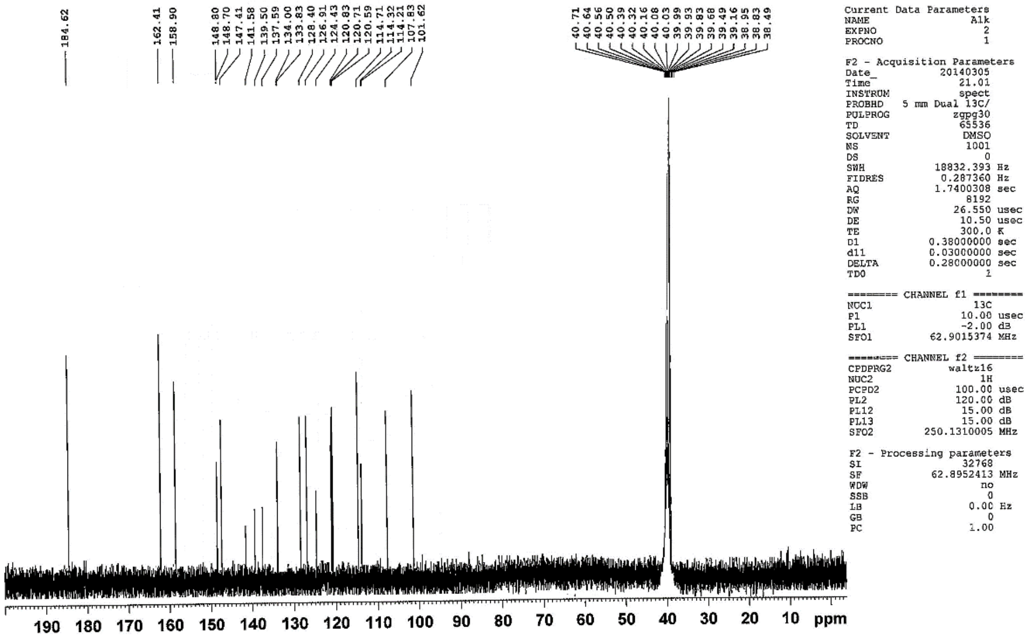

**Compound 14**: $^{1}$H and $^{13}$C NMR

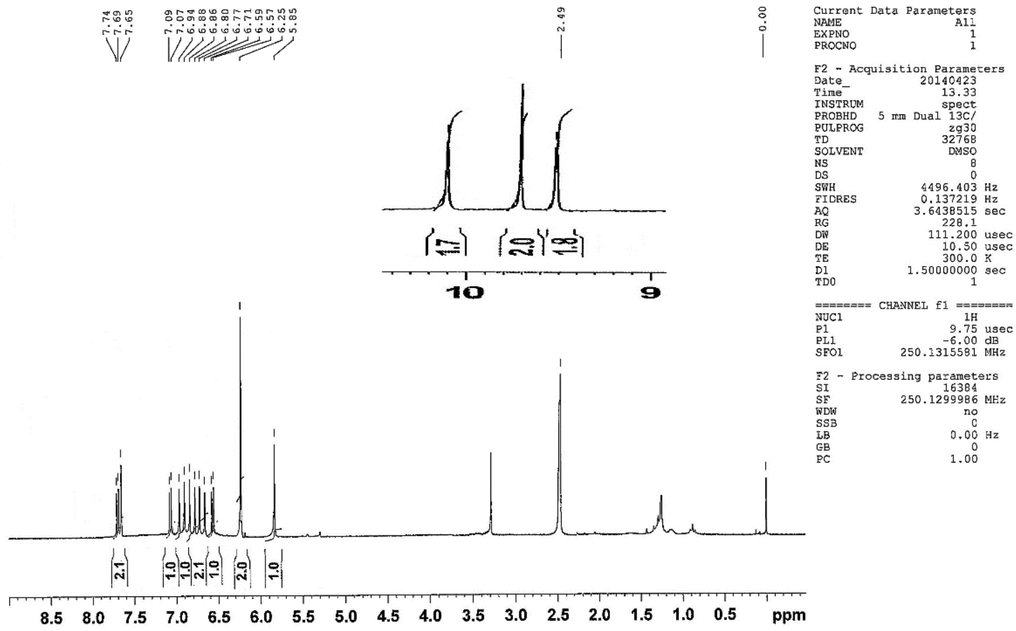

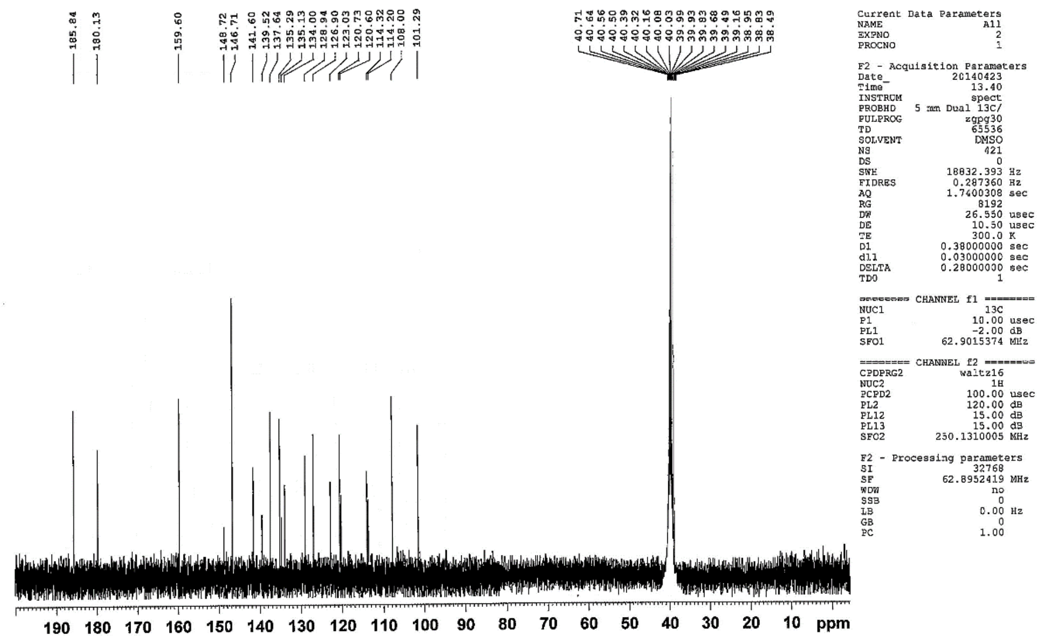

**Compound 15**: $^{1}H$ and $^{13}C$ NMR

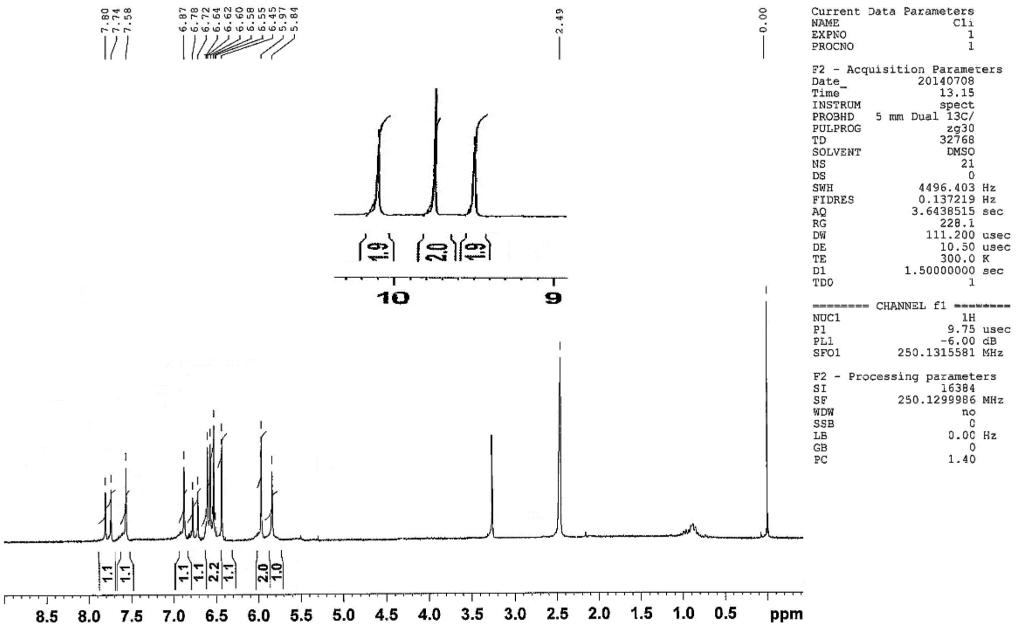

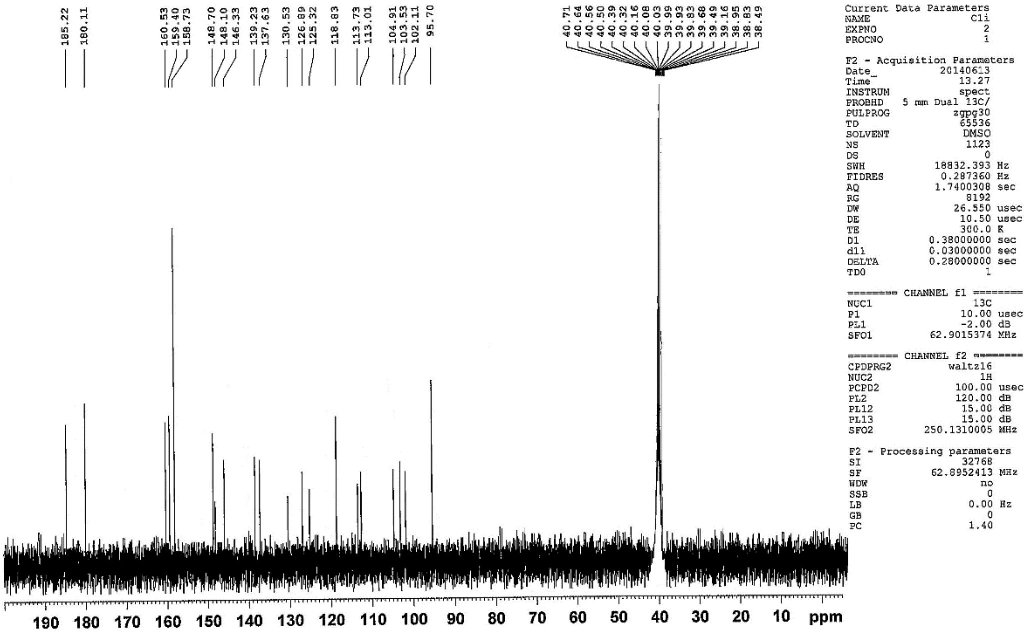

**Compound 16**: $^{1}$H and $^{13}$C NMR

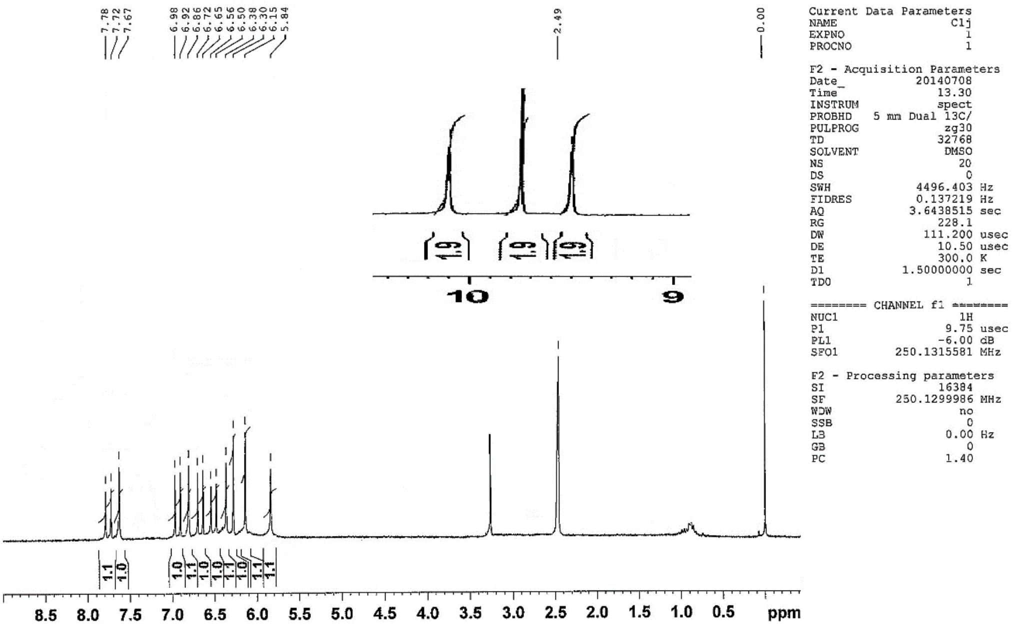


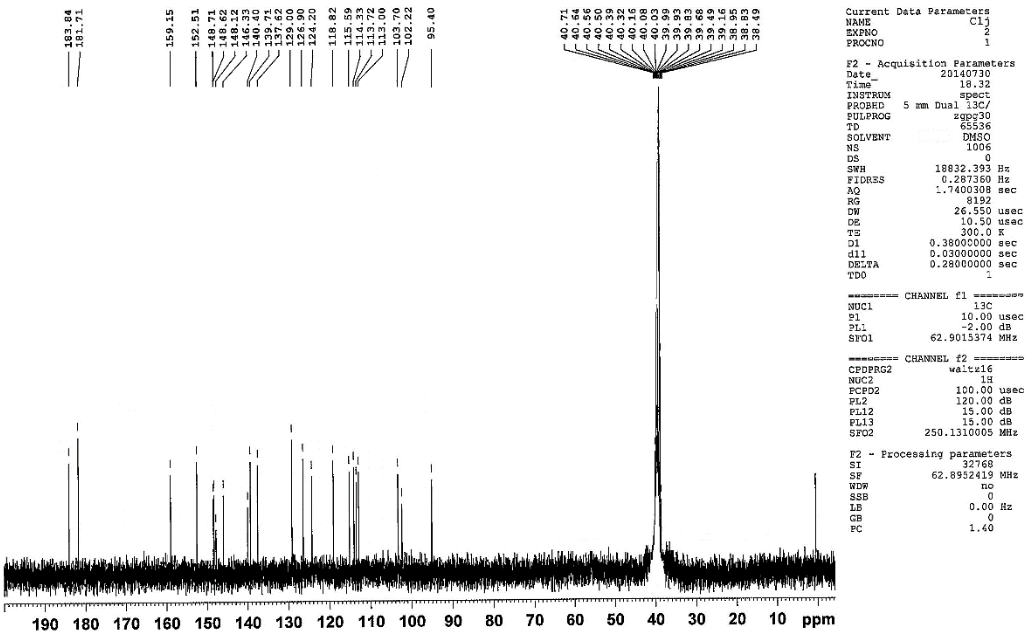

**Compound 17**: $^{1}$H and $^{13}$C NMR

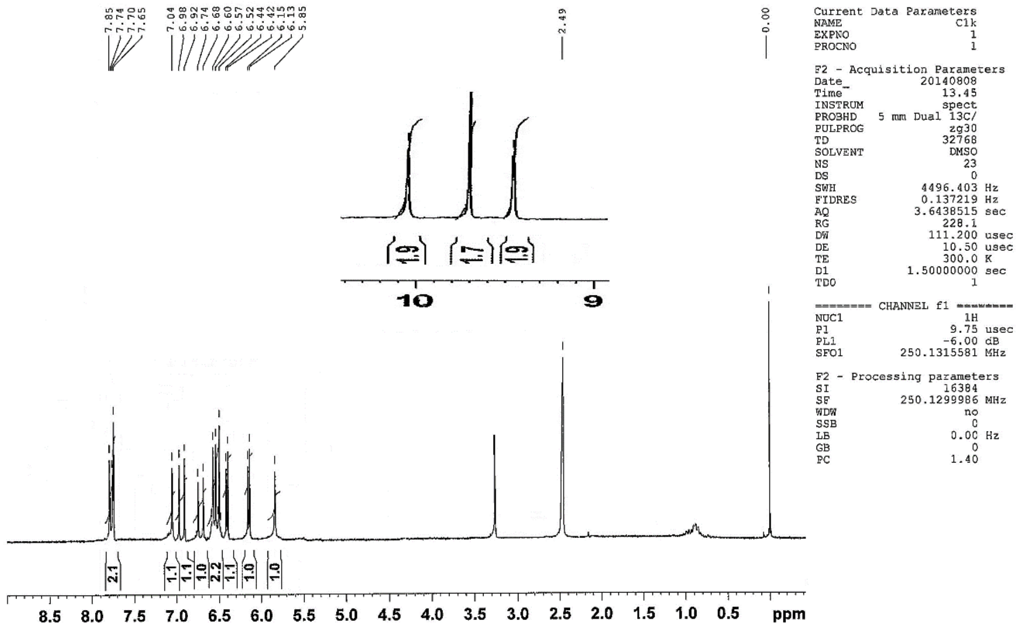


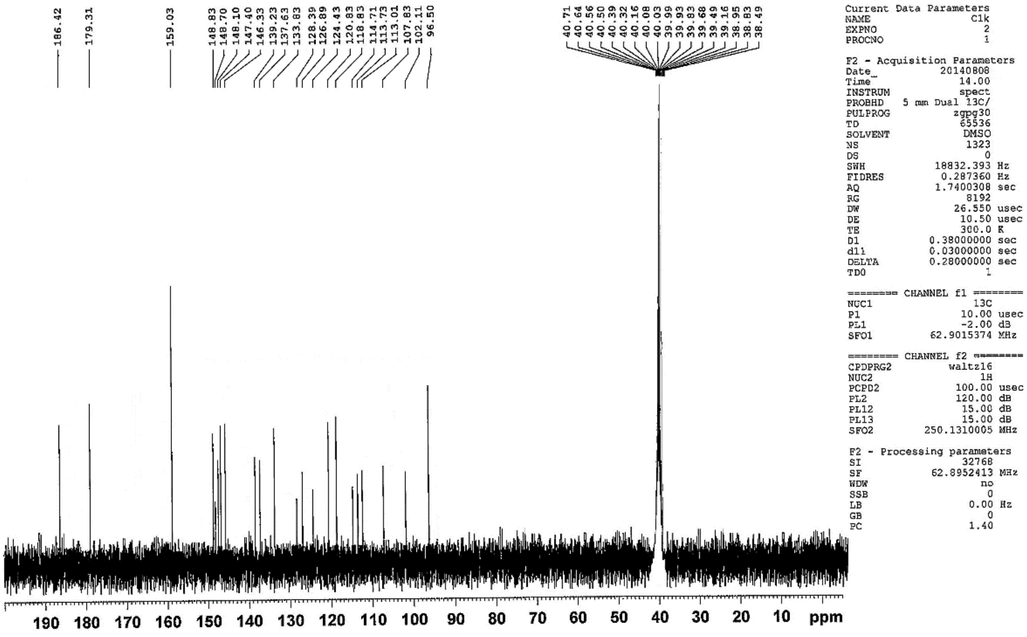

**Compound 18**: $^{1}$H and $^{13}$C NMR

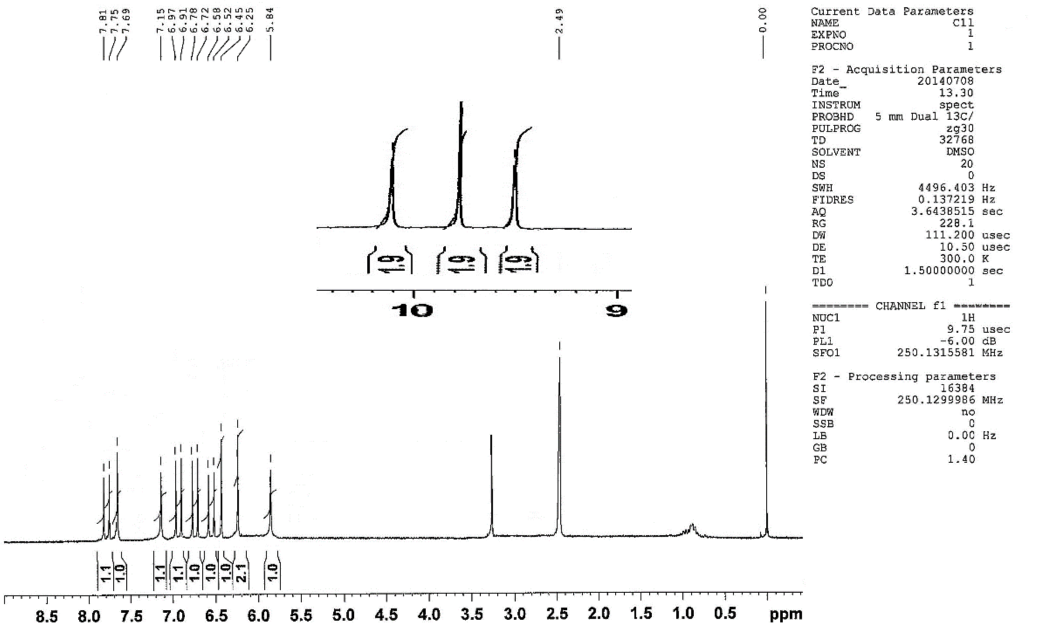

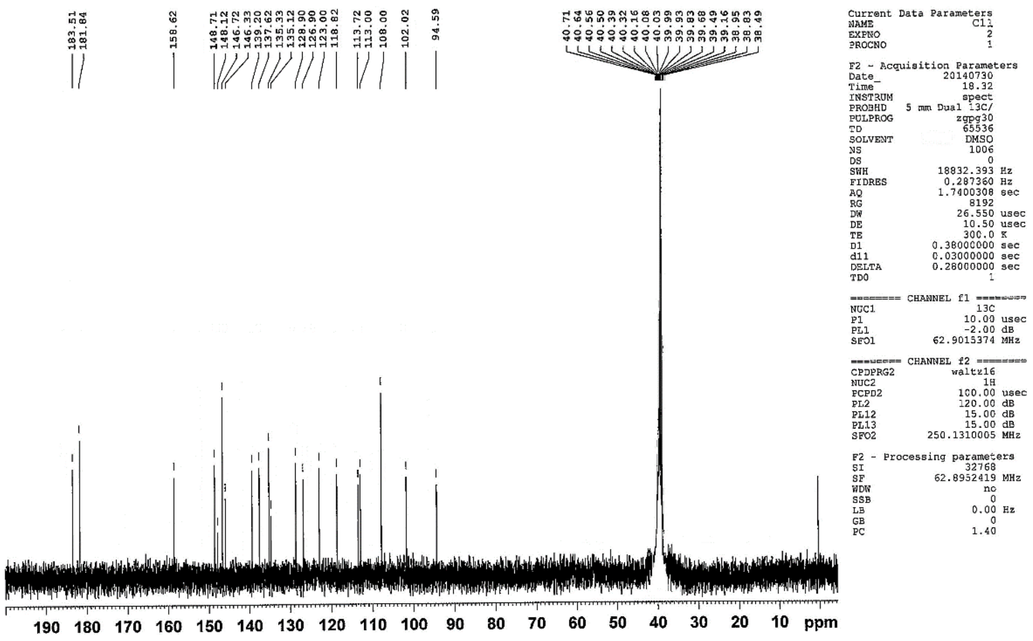

Supplement: Supplementary file 1 [file molecules-26-04550-s001.zip › molecules-1267907-supplementary.pdf]
